# From Research Trend to Performance Prediction: Metaheuristic-Driven Machine Learning Optimization for Cement Pastes Containing Bio-Based Phase Change Materials

**DOI:** 10.3390/polym17182541

**Published:** 2025-09-19

**Authors:** Leifa Li, Wangwen Sun, Lauren Y. Gómez-Zamorano, Zhuangzhuang Liu, Wenzhen Zhang, Haoran Ma

**Affiliations:** 1 Xinjiang Jiaotou Construction Management Co., Ltd., Urumchi 830000, China; 2 School of Highway, Chang’an University, Xi’an 710064, China; 3 Programa Doctoral en Ingeniería de Materiales, Facultad de Ingeniería Mecánica y Eléctrica, Universidad Autónoma de Nuevo León, Ave. Universidad s/n, Ciudad Universitaria, San Nicolás de los Garza 66455, Nuevo León, Mexico; 4 Key Laboratory of Special Area Highway Engineering, Ministry of Education, Xi’an 710064, China; 5 International Joint Laboratory for Sustainable Development of Highway Infrastructures in Special Regions, Xi’an 710064, China

**Keywords:** bio-based phase change materials, cementitious composites, performance prediction, literature visualization analysis, model optimization, sustainability

## Abstract

This study presents an integrated approach combining bibliometric analysis and machine learning to explore research trends and predict the performance of cement pastes containing bio-based phase change materials. A bibliometric review of 5928 articles from the Web of Science Core Collection was conducted using CiteSpace (v.6.3.R1) to identify research hotspots. A dataset of 100 experimental samples was compiled, including nine input variables and three output properties identified as thermal conductivity (Tc), latent heat capacity (LH) and compressive strength (CS). Four machine learning algorithms (SVR, RF, XGBoost, and CatBoost) were optimized using five metaheuristic algorithms (GA, PSO, WOA, GWO, and FFA), resulting in 24 optimized hybrid models. Of all the models considered, CatBoost-WOA achieved the best overall performance, with R^2^ values of 0.927, 0.955, and 0.944, and RMSEs of 0.0057 W/m·K, 1.84 J/g, and 2.91 MPa for Tc, LH, and CS. Additionally, SVR-GWO and XGBoost-WOA also showed strong generalization and low error dispersion. The developed models provide a transferable and data-driven modeling pipeline for predicting the coupled thermal and mechanical behavior of cement pastes containing bio-based phase change materials.

## 1. Introduction

Green building materials play a vital role in promoting sustainability in the construction industry by minimizing environmental impact, conserving resources, and reducing energy consumption [1,2,3]. With the advancement of global carbon neutrality goals, bio-based phase change materials (BPCMs) have attracted growing attention due to their renewability and excellent thermal regulation capability [2]. These materials can absorb and release latent heat during phase transitions, thereby regulating the internal temperature of buildings and significantly improving energy efficiency [3]. The interaction between BPCMs and cementitious materials has emerged as a central focus in green energy engineering research. The building heat island (BHI) effect may occur when the internal temperature is much higher than the ambient temperature. This effect is closely related to the thermal properties of construction materials [4]. Due to their high thermal capacity and conductivity, cement-based materials significantly contribute to this effect. Previous studies have shown that incorporating BPCMs into cementitious systems allows excess daytime heat to be absorbed and gradually released at night, effectively narrowing the diurnal temperature range [4,5]. This regulation is primarily attributed to the latent heat storage and temperature sensitivity characteristics of BPCMs. Currently, BPCMs applied in cement-based materials include both low-molecular-weight compounds (e.g., fatty acids and waxes) and polymeric substances (e.g., bio-based PEGs or biopolymers), depending on the application [6]. These materials reduce environmental impact and enable the reuse of biomass-containing waste, thereby improving resource efficiency. However, their application in cementitious materials still encounters challenges, including poor interfacial compatibility with cement paste, insufficient thermal cycling stability, and the high cost of encapsulation and production [6,7]. Current research focuses on four main aspects: (1) Development of novel BPCMs with improved thermal properties; (2) Prevention of material leakage via microencapsulation or porous carrier loading techniques; (3) Improvement of overall durability, with particular emphasis on the interfacial bonding strength between BPCMs and the cement pastes; and (4) Quantitative evaluation of improvements in thermal and mechanical performance under practical application conditions.

Although progress has been made in these areas, a systematic analysis of the research landscape, key technological breakthroughs, and emerging trends is still lacking. To fill the gap, the study employs a bibliometric approach based on the Web of Science database to analyze the research developments on BPCM-integrated cementitious materials over the past twelve years [7,8,9]. Researchers have developed a range of innovative cementitious composites incorporating BPCMs. In practical applications, these materials have demonstrated promising thermoregulatory performance. For example, several studies have shown that incorporating 20% coconut oil-derived BPCMs into cement mortar can reduce surface temperatures by 5–8 °C and decrease energy consumption by up to 30%. Thermal stability is a critical factor in the engineering application of BPCMs, especially considering that the hydration heat in cement-based materials can reach 70–80 °C [10,11]. Thermogravimetric analysis confirms that most plant oil-based BPCMs remain stable below 100 °C, while sugar alcohol-based BPCMs show superior thermal resistance. To mitigate leakage during phase change, two common strategies are employed: polymer microencapsulation and loading onto porous supports [12,13]. To improve the representativeness of our trend analysis, we additionally considered recent comprehensive review studies [12,13,14,15,16], which highlight the growing interest in sustainable cementitious composites incorporating bio-based PCMs. These reviews emphasize evolving research focus from thermal performance control to multi-functional integration, intelligent modeling, and the application of AI-assisted design. By incorporating these perspectives, our bibliometric analysis is placed in a broader context, providing a more complete overview of current research trends and methodological developments in the field.

Further, incorporating 5% graphene has been shown to significantly enhance the thermal conductivity of BPCM cement pastes [14], increasing thermal response rate by 40%. However, the addition of BPCMs tends to reduce the compressive strength of cementitious materials by 10–20%. This drawback can be mitigated through optimization of aggregate gradation and the use of active additives such as nano-silica [15,16,17]. Dynamic mechanical analysis and three-point bending tests have confirmed that surface-treated BPCMs can improve fracture toughness by up to 12%, offering both thermal and mechanical benefits [18]. These findings lay a solid foundation for the practical implementation of BPCMs in sustainable construction. The performance of BPCM cement pastes is influenced by multiple interacting factors, including PCM type and dosage, water-to-cement ratio, porosity, ambient temperature/humidity, and microstructural evolution [19,20]. Traditional experimental methods for thermal performance assessment are costly, time-consuming, and often fail to capture complex nonlinear interactions between variables. As a result, data-driven prediction models have gained traction in recent years [21,22].

Machine learning (ML) techniques have shown excellent capabilities in nonlinear regression and robust handling of complex data structures [23,24]. The study has demonstrated the value of AutoML-enhanced approaches in predicting the behavior of cementitious composites, reinforcing the role of machine learning in material performance analysis [24]. They are now widely used for prediction in cement-based materials. Compared to conventional regression methods, ML algorithms achieve superior performance in capturing intricate relationships between material composition, mix proportions, curing conditions, and target properties such as thermal conductivity, latent heat, and compressive strength [25,26]. Several supervised learning models have been applied in the domain. Support Vector Regression (SVR) is effective for small-sample learning scenarios [27]. Random Forest (RF), a bagging-based ensemble model, helps reduce overfitting and improve generalization performance. Gradient Boosting frameworks such as XGBoost and CatBoost have demonstrated high accuracy and efficiency in handling nonlinear feature interactions and high-dimensional data [28,29]. To further improve predictive accuracy, metaheuristic optimization algorithms such as Particle Swarm Optimization (PSO), Whale Optimization Algorithm (WOA), Grey Wolf Optimizer (GWO), and Firefly Algorithm (FFA) have been employed for hyperparameter tuning [30,31]. These nature-inspired algorithms mimic collaborative search behaviors to perform global optimization, thereby improving both model accuracy and robustness. In this study, four machine learning models (SVR, RF, XGBoost, and CatBoost) were developed to predict the thermal and mechanical performance of BPCM-cement composites, using 100 experimental datasets. These models were optimized using various metaheuristic algorithms. Model performance was evaluated using RMSE, MAE, and R^2^ metrics. Feature importance was further analyzed to identify the dominant influencing factors and to support material design optimization [32,33,34].

In summary, this work establishes a systematic modeling framework for high-precision prediction of the thermal performance of BPCM cement pastes. By integrating four representative machine learning models with multi-strategy metaheuristic optimization algorithms (GA, WOA, GWO, and FFA), the proposed framework improves predictive accuracy and improves adaptability to complex material systems [35,36]. This approach provides a solid theoretical and technical foundation for intelligent modeling of BPCM-integrated green building materials and shows strong potential for large-scale engineering applications.

## 2. Techniques and Methodology

This study employs bibliometric analysis to comprehensively examine current research trends on the thermal performance of cement pastes incorporating BPCMs. The methodological framework is divided into three stages: literature retrieval, data mining, and bibliometric analysis. In the first stage, a structured and topic-focused dataset was established by selecting relevant databases and formulating precise search strategies. The second stage involved the screening and cleaning of the retrieved literature to ensure the completeness and accuracy of the final dataset. In the third stage, CiteSpace software was employed to conduct knowledge mapping and visualization. To systematically address the core issues in this field, four key research questions were proposed: (1) What are the thematic evolution trends in research on the thermal and mechanical properties of BPCM-integrated cementitious materials? (2) Which high-frequency terms and keywords dominate the existing co-occurrence networks? (3) What is the global distribution of leading research institutions and countries in this field, and are there any significant patterns of international collaboration? (4) Which authors, institutions, and journals exhibit high productivity and academic influence?

By answering these questions, this study seeks to identify the technical hotspots and emerging research frontiers in the field. The findings are expected to offer theoretical insight and strategic direction for the future development and application of BPCM-cement composites in building thermal regulation and energy-efficient design.

### 2.1. Bibliometric Analysis

#### 2.1.1. Literature Retrieval

Literature was collected from the Web of Science Core Collection (WoS) due to its broad and reliable coverage of engineering, construction materials, and sustainable building technologies [37]. WoS provides well-structured academic metadata, including author affiliations, institutional data, citations, journal metrics, and country-level information. It also offers extensive indexing of leading journals related to green materials, phase change materials (PCMs), and energy-efficient construction, ensuring both representativeness and data consistency [38,39]. During the search and data processing, minor refinements and iterative adjustments were applied to optimize the dataset for the purposes of this study, ensuring accuracy and consistency throughout.The search query used was: TS = (“phase change materials” OR “bio-based PCM” OR “cementitious materials” OR “energy efficiency” OR (“green building materials” OR “low-carbon materials” OR “renewable building materials”) AND “thermal performance”). The search was limited to articles, reviews, conference papers, and early access publications from 2013 to 2024.

Keywords were selected based on core concepts in green materials and energy-efficient construction, with terminology standardized according to international conventions on PCMs, cementitious materials, and thermal properties to ensure consistent term matching and accurate retrieval [40,41]. All retrieved records were subjected to duplicate removal, format normalization, and keyword standardization, resulting in a clean and unified research dataset [42,43,44]. During the search and data processing, minor refinements and iterative adjustments were applied to optimize the dataset for the purposes of this study, ensuring accuracy and consistency throughout. A total of 7214 records were collected from the WoS Core Collection, with 612 duplicates and non-English records removed, and 674 irrelevant studies excluded, leaving 5928 relevant publications. WoS was chosen for its comprehensive coverage of materials science and construction engineering, ensuring standardized metadata for reliable bibliometric analysis [45,46].

#### 2.1.2. Publication Trends

Bibliometric data from the Web of Science Core Collection show a significant upward trend in publications since 2013. In particular, the topic of thermal performance in cementitious composites incorporating BPCMs has gained increasing academic attention over the past decade.

**Figure 1:**
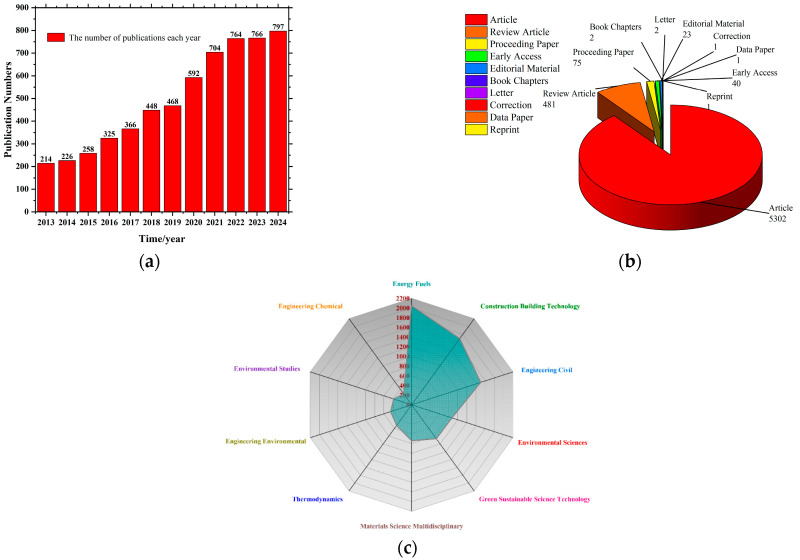
Bibliometric analysis of research on bio-based phase change materials. (**a**) Annual publication trends; (**b**) Distribution of document types; (**c**) Disciplinary subject classification.

As shown in Figure 1a, the annual number of publications between 2013 and 2024 demonstrates the steady growth trajectory, with red bars indicating the yearly output. In the early stage (2013–2015), annual publications were modest, between 200 and 300. Since 2016, a marked increase in research activity is observed, with the annual number surpassing 350. Key inflection points occurred in 2015 and 2020. The number of publications first exceeded 250 in 2015 and then surpassed 550 in 2020. This growth reflects increasing global investment in green building materials, particularly BPCMs. Since 2021, the average annual output has remained consistently above 700 publications, reaching an all-time high of 797 articles in 2024. The trend highlights the growing role of BPCMs in low-carbon construction and energy-efficient design. It also points to the rise of thermal energy storage technologies and cement-based heat regulation systems as central topics [47].

Figure 1b illustrates the distribution of publication types. Journal articles account for the vast majority (89%), followed by review papers (8%), with other formats contributing marginally. This distribution indicates that the field is mainly driven by original research and experimental studies with strong engineering relevance [48].

Thematic analysis reveals an evolution of focus over time. In the early years, most studies were centered on basic thermal characterization of bio-based PCMs [49]. In recent years, research has shifted toward composite material design, thermal performance evaluation, and mechanistic understanding of phase transition behavior. Moreover, increasing attention is being paid to thermal conductivity control, interfacial heat transfer, and other advanced topics, reflecting a deepening and systematization of research in this domain. In parallel, the interdisciplinary scope of the field has expanded significantly [50,51]. Earlier studies were concentrated within materials science and architectural engineering. However, recent publications demonstrate growing integration with environmental science, energy systems, civil engineering, and even artificial intelligence. The shift suggests the emergence of a multidisciplinary research network centered on bio-based PCMs, encompassing thermal energy engineering, microstructural design of cementitious systems, and intelligent performance prediction [52,53].

In summary, over the past twelve years, the field of bio-based PCM–cement composites has witnessed a rapid growth in scholarly output, underscoring its scientific significance and wide-ranging potential in the development of next-generation green building materials.

#### 2.1.3. Knowledge Network Analysis

This study investigates collaborative patterns and knowledge diffusion in cementitious composites incorporating bio-based phase change materials, using a multi-dimensional bibliometric network analysis. By mapping authorship, institutional affiliations, national collaborations, co-citation structures, we reveal the distribution of academic resources, research impact, and interdisciplinary innovation mechanisms [54]. Results demonstrate the emergence of a multi-layered collaborative network centered on high-productivity authors, influential institutions, and core journals, exhibiting distinct clustering tendencies, geographical linkages, and cross-disciplinary integration.

(1) Country Collaboration Network

**Figure 2:**
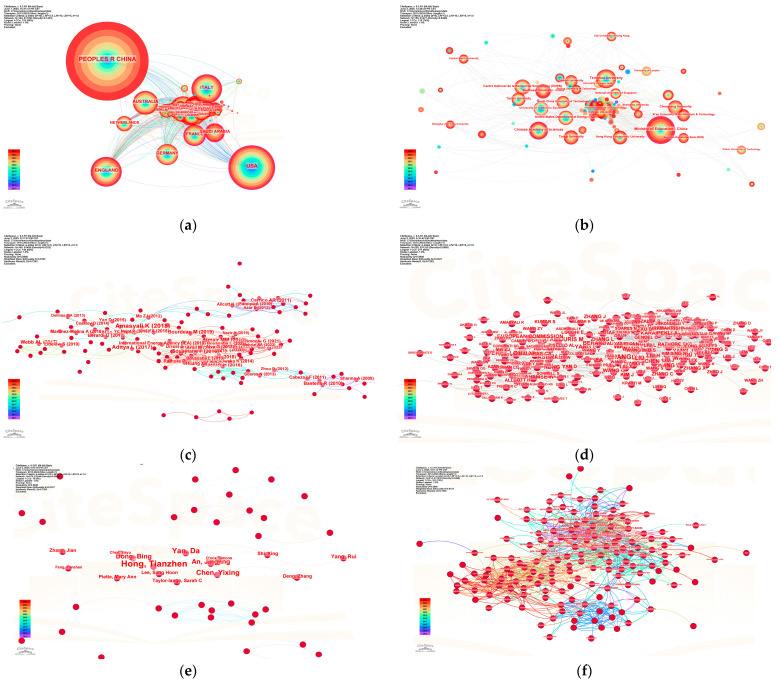
Knowledge mapping analysis of research on bio-based phase change materials. (**a**) Country collaboration network; (**b**) Institutional collaboration network; (**c**) Author collaboration network; (**d**) Author co-citation network; (**e**) Highlighted core author network; (**f**) Journal co-citation network.

As depicted in Figure 2a, China leads the field with the largest node size, highest annual publication output, and citation frequency, highlighting its strong research capacity and significant global influence. The United States, Malaysia, Italy, and Iran serve as secondary hubs and exhibit strong collaborative ties with China, particularly evident in the China–U.S. and China–Malaysia partnerships as indicated by the thickened edges in the network. European countries such as Italy, the United Kingdom, Germany, and France form a central cluster characterized by moderate node sizes but high interconnectivity, reflecting well-established regional research networks. Emerging economies like Iran and Egypt exhibit expanding nodes, signaling growing research engagement.

(2) Institutional Collaboration Network

Figure 2b highlights a China-centric framework, where universities (China University of Mining and Technology, Tsinghua University, Southeast University, Shenzhen University, Tongji University, South China University of Technology) occupy densely connected core positions. Cross-border collaborations with Universiti Teknologi Malaysia, Iran University of Science and Technology, and Università di Pisa are evident, though the overall network remains sparse, suggesting untapped potential for institutional synergy.

(3) High-Impact Literature and Knowledge Diffusion

As depicted in Figure 2c, the document co-citation network identifies seminal works by Ammar Yahia (2016), Kheradmand (2018), and Khudhair (2004) as persistent knowledge anchors in BPCM thermoregulation and composite design. The stable expansion of this network indicates a mature yet dynamically evolving knowledge base.

(4) Author Co-Citation Analysis

Figure 2c,d identify Chinese scholars such as Hong Tianzhen, Yang Da, Zhang Yuhan, and Zhou Bing as central nodes who are frequently co-cited for their contributions to building energy efficiency, PCM heat transfer modeling, and computational optimization. Their work forms the primary knowledge conduit, flanked by international researchers (Petithuguenin, Taylor Hogue, Feng Qian), illustrating a China-led yet globally interconnected citation structure. Peripheral low-frequency authors denote emerging research groups.

(5) Author Collaboration Network

There are tight-knit teams in the network, such as the collaboration between Hong Tianzhen and Yang Da. However, Figure 2e reveals a low global network density, with numerous fragmented small clusters. This indicates limited cross-team integration beyond institutional boundaries.

(6) Journal Co-Citation Analysis

Figure 2f identifies the core journals in this research domain as *Construction and Building Materials*, which functions as a hub node with 736 co-citations, followed by *Renewable and Sustainable Energy Reviews*, *Journal of Cleaner Production*, and *Energy and Buildings*. The expanding nodes in *Sustainability*, *Applied Energy*, and the *Journal of Thermal Analysis and Calorimetry* reflect a disciplinary convergence toward environmental management and energy systems.

Studies have increasingly focused on the thermal and mechanical performance of cementitious composites incorporating bio-based phase change materials (BPCMs). Materials such as stearic acid, palmitic acid, plant waxes, and PEG compounds have been evaluated for their latent heat capacity, thermal conductivity, and compressive strength after incorporation into cement matrices. These BPCMs offer dual functionality by enabling heat storage while maintaining acceptable structural integrity. However, challenges such as dispersion, compatibility, and thermal stability remain critical factors influencing their practical application.

### 2.2. Evolution of Research Trends

Recent research on bio-based PCM-integrated cementitious composites has predominantly focused on the precise characterization of thermal-physical parameters, such as thermal conductivity, latent heat, and phase change temperature. In recent years, emerging terms such as “bio-based PCM”, “thermal regulation”, and “machine learning modeling” have gained marked prominence, highlighting a shift in the field from traditional material development toward intelligent modeling and multi-functional system integration. Further analysis reveals the increasing attention in exploring the interrelationship between composite performance and key durability and microstructural properties. This trend underscores an evolving research focus toward understanding the coupled behavior of thermal, mechanical, and structural parameters, which is critical for advancing the practical application of BPCM cement pastes in sustainable construction systems.

#### 2.2.1. Main Terms Analysis

To comprehensively analyze research hotspots and thematic evolution of bio-based phase change materials in cementitious building materials, this study employed CiteSpace to conduct a keyword co-occurrence analysis on core publications from 2013 to 2024 [55,56]. The keyword analysis was conducted using CiteSpace, a bibliometric software that generated co-occurrence and clustering results from the Web of Science Core Collection.

**Figure 3:**
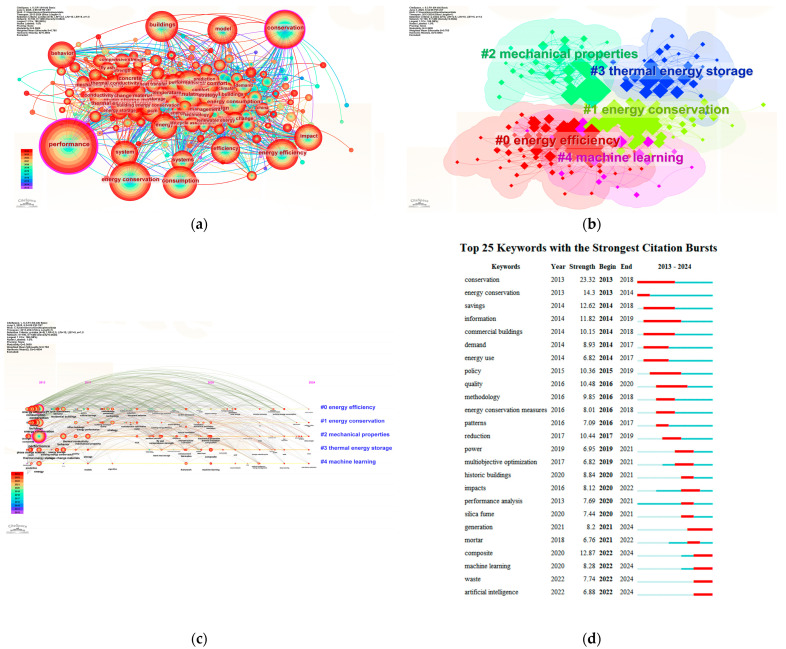
Analysis of research hotspots and evolutionary trends. (**a**) Keyword co-occurrence and burst detection; (**b**) Evolution of research hotspots over time; (**c**) Topic clustering and development path mapping; (**d**) Keyword clustering, burst detection, and temporal evolution analysis.

As shown in Figure 3a, high-frequency keywords such as “performance”, “model”, “energy efficiency”, “thermal conductivity”, and “conservation” form the dense core of the co-occurrence network. The size and color of each node represent keyword frequency and temporal span, respectively, with a color gradient from cool to warm indicating the chronological evolution of research focus. The dense interlinkages between nodes reflect strong semantic and thematic associations, constituting the intellectual foundation of the field [57,58,59]. The co-occurrence network visualizes the connections and clustering of prominent research topics. Larger nodes like “performance”, “model”, “energy conservation”, and “thermal conductivity” indicate concentrated academic attention and high relevance.

As shown in Figure 3b, the clustering algorithm segmented the keywords into five major thematic modules, labeled #0 through #4.

Cluster #0: “Energy Efficiency”–covering topics such as building energy saving, PCM-based composites, and thermal regulation mechanisms;

Cluster #1: “Energy Conservation”–emphasizing energy-saving strategies and holistic energy management, representing a core research trajectory over the past decade;

Cluster #2: “Mechanical Properties”–focusing on the impact of PCM incorporation on cementitious material strength, including compressive strength and elastic modulus;

Cluster #3: “Thermal Energy Storage”–addressing latent heat behavior, energy storage efficiency, and thermal cycling stability, central to PCM research;

Cluster #4: “Machine Learning”–signifying the rise of data-driven modeling and the increasing role of artificial intelligence in performance prediction and material optimization.

Figure 3c reveals the emergence and development of these clusters over time. Long-standing core topics such as “energy efficiency” and “energy conservation” have remained active since 2013. In contrast, emerging keywords such as “machine learning” and “artificial intelligence” have shown rapid growth since 2020, indicating a shift toward intelligent modeling and interdisciplinary integration. The research focus has evolved from early concerns such as “energy conservation”, “thermal conductivity”, and “heat transfer”, toward more nuanced topics including “microstructure”, “composite materials”, “optimization”, and “machine learning”, demonstrating the field’s increasing complexity and depth. Figure 3d presents the keyword burst detection results from 2013 to 2024. Burst strength indicates a sharp rise in keyword citation frequency within a specific period, signaling high attention at that time. For example, “conservation” showed a burst strength of 23.23 during 2013–2018, marking it as a foundational research term in the early stage. Similarly, “energy conservation” and “commercial buildings” showed significant surges between 2014–2018 and 2015–2019, respectively, reflecting the strong relevance of energy-saving strategies and real-world application contexts. Since 2020, emerging terms such as “machine learning”, “waste”, and “artificial intelligence” have become increasingly active, indicating a shift from conventional experimental evaluation to interdisciplinary approaches incorporating intelligent prediction, low-carbon material design, and system-level performance analysis.

Overall, the keyword landscape shows a clear trajectory: research has expanded from unidimensional thermal performance and energy storage studies toward multidimensional investigations of mechanical compatibility, microstructural control, and intelligent optimization [60]. While “energy efficiency” and “thermal storage” remain dominant themes, urban thermal environment responses have not yet formed a concentrated research cluster, highlighting opportunities for future work on engineering feasibility and multi-scale performance evaluation [18,60,61].

#### 2.2.2. Performance Evaluation of Bio-Based Phase Change Materials

Bio-based phase change materials (BPCMs) are environmentally friendly and renewable thermal-regulating materials. They include fatty acids, natural waxes, vegetable oil derivatives, and biomass-derived polyethylene glycols (PEGs) [62]. Among them, fatty acid-based PCMs are particularly valued in high-temperature cement composite applications due to their high melting points and large enthalpy of fusion [63]. In particular, PEGs derived from biomass-based feedstocks, have been widely studied in green building applications owing to their excellent specific heat capacity, low toxicity, chemical stability, and biodegradability during phase transition [64]. These materials enable controlled thermal buffering during solid–liquid transitions, making them a preferred functional medium in low-carbon cementitious systems.

Particle size is a key parameter affecting the thermal efficiency and leakage control of BPCMs in cement-based matrices [65]. The particle size of BPCMs varies depending on the form, ranging from microencapsulated particles (~0.2 μm) to bulk granules (~9.5 mm), with average sizes for practical applications around 0.4–1 mm. For PEG2000 and PEG4000, the typical particle sizes are about 0.4 mm and 0.6 mm, respectively. Owing to their high specific surface area, smaller particles promote homogeneous dispersion in the cementitious matrix and improve thermal responsiveness and heat conduction. These fine particles facilitate rapid thermal exchange within porous matrices but also pose a greater risk of leakage, especially in unencapsulated systems or those lacking effective gelation [38,39]. In contrast, larger particles reduce leakage risk but may suffer from uneven distribution and reduced thermal regulation efficiency. Therefore, optimal particle size must be determined based on absorbability, porosity, permeability, and encapsulation efficiency to balance heat transfer performance with leakage control.

Among thermal performance parameters, melting point and enthalpy of fusion are the most representative indicators for bio-based PCMs [66]. The melting point defines the effective temperature range for phase transition and serves as a fundamental parameter in the design of PCM-enhanced thermal regulation systems. Low melting point PCMs may fail prematurely in high-temperature environments, while PCMs with excessively high melting points may not undergo phase change under typical building conditions. The melting points of bio-based PCMs range from 3 °C to 80 °C, averaging 46.67 °C, which aligns well with typical surface temperature fluctuations in building components. For internal thermal stabilization, PCMs with melting points between 45–60 °C are generally more appropriate [53,66]. Several bio-based PCMs such as stearic acid, plant waxes, and PEG compounds exhibit excellent heat storage capacities. These materials were selected based on their high latent heat capacities, suitable melting points, and chemical compatibility with cementitious matrices. Stearic acid and palmitic acid are long-chain saturated fatty acids typically derived from plant oils. They exhibit melting points of approximately 69 °C and 63 °C, respectively, and possess high latent heat storage capacities (~210 J/g and ~195 J/g). Their relatively sharp melting transitions and good thermal stability make them effective for temperature regulation in building applications. However, their hydrophobic nature and limited dispersibility in aqueous environments may hinder uniform integration into cement matrices. Plant wax, primarily composed of natural esters, fatty alcohols, and long-chain hydrocarbons, offers a broader phase transition range (~60–70 °C) and exhibits moderate thermal stability. Its semi-solid state at room temperature reduces the risk of leakage during phase transition, and its bio-based origin supports sustainability goals. PEG 6000, a bio-derived polyether compound, was included as a polymeric PCM due to its melting point (~61 °C) and latent heat (~170 J/g). To improve its stability and prevent leakage during thermal cycling, PEG 6000 was applied in a microencapsulated form. The encapsulation process involved enclosing the PCM in a polymeric shell, which improved dispersion, interfacial bonding, and retention within the cementitious matrix.

For instance, stearic acid offers an enthalpy range of 129.6–221.6 J/g and melting points between 40–80 °C, combining high energy storage with thermal stability, which makes it suitable for high heat-load building structures [67]. Similarly, PEG4000 and PEG2000 possess latent heat values of 135.2 J/g and 180 J/g, respectively. Although their thermal capacity is lower than that of stearic acid, their superior compatibility, low toxicity, and good processability make them mainstream candidates for integration into cementitious systems [68]. By contrast, some PCMs like n-tetradecane or palmitic acid offer high latent heat but have melting points below the operating range of most cement-based elements, limiting their applicability in construction [69].

Beyond thermal parameters, the physicochemical compatibility between BPCMs and cementitious matrices must also be considered [70]. Issues such as acidic degradation, thermal expansion mismatch, and poor dispersion may lead to mechanical property deterioration. High-enthalpy PCMs are more suitable for applications with large diurnal temperature variations and high peak thermal loads, such as exterior building envelopes. Medium-enthalpy, high-stability PCMs are more appropriate for internal mortar-based thermal regulation layers [71]. When appropriately selected, these materials enable passive thermal regulation that reduces energy peak demands and delays heat flux transmission within structural elements [72,73]. Despite their promising energy storage potential and environmental benefits, the practical application of BPCMs in cement systems still faces multiple challenges, such as leakage control, thermal degradation, and inconsistent dispersion [74,75]. Selection based solely on thermal performance is insufficient; instead, a holistic evaluation involving particle size, interface compatibility, and service durability is essential. Currently, most investigations remain at the laboratory scale, and standardized testing protocols or life-cycle performance evaluations for thermal–mechanical synergy in real-world cement matrices are still lacking [76]. Therefore, both the intrinsic thermal behavior and interfacial interactions with the cementitious matrix jointly determine the feasibility of BPCMs in energy-efficient construction [77,78].

#### 2.2.3. Analysis of Thermal and Mechanical Characteristics in Cement Pastes with Different BPCMs

Integrating bio-based phase change materials into cementitious materials requires a careful balance between optimizing thermal performance and maintaining mechanical stability [79,80]. However, the incorporation of BPCMs can have multifaceted impacts on the mechanical properties of cement paste. Studies have shown that increasing PCM dosage often leads to reductions in compressive strength and splitting tensile strength. The degradation is primarily attributed to the low elastic modulus of BPCMs compared to cement hydration products. In addition, PCM particles tend to induce microvoids or interfacial debonding, thereby compromising matrix densification and structural integrity [81,82]. The negative impact becomes more pronounced when the particle size of BPCMs exceeds 1 mm, due to poor dispersion and localized stress concentration. In contrast, microencapsulated BPCMs with smaller particle sizes exhibit improved interfacial compatibility and uniform distribution within the matrix, which mitigates mechanical deterioration [83,84]. Although overall strength is reduced, some mechanical parameters improve with PCM incorporation [85]. BPCM-modified mortars also show enhanced durability under repeated thermal–moisture cycling, with higher resistance to thermal fatigue than conventional mixes [86,87].

From the other perspective, the type of BPCM plays a decisive role in determining the heat regulation efficiency. Commonly used PEG-based PCMs (e.g., PEG2000, PEG4000) have melting points close to the peak surface temperatures of cementitious elements under solar exposure, allowing for precise heat release during high-temperature periods [88]. Experimental results indicate that incorporating PEG-based BPCMs can lower internal core temperatures by approximately 7–9 °C under intense solar radiation. In addition, they significantly reduce heat flux propagation, enabling a delayed peak thermal response [89]. The optimal dosage of BPCMs remains a key research focus. Most studies suggest a threshold of approximately 14 wt%, which achieves a favorable balance between thermal regulation and mechanical performance, with limited compromise in structural strength [90].

However, the type, encapsulation method, and dispersion technique of BPCMs significantly influence both thermal and mechanical behavior. For instance, high-purity fatty acid PCMs, while exhibiting high latent heat values, may undergo alkaline degradation in the cementitious environment, adversely affecting interfacial structure [91]. Microencapsulated PEGs demonstrate superior chemical stability and compatibility, making them more suitable for engineering applications. In contrast, microencapsulated bio-based phase change materials (BPCMs), such as PEG 6000, exhibit superior thermal stability and improved compatibility with cementitious materials. The PEG was microencapsulated using industrial spray-drying, forming spherical particles with an average diameter of approximately 10 to 20 μm, as provided by the manufacturer. This encapsulation process creates a polymeric shell that effectively isolates the PCM core from the alkaline cement matrix, thereby preventing leakage, reducing chemical degradation, and enhancing interfacial bonding with the hydration products. The smaller particle size also facilitates uniform dispersion in the cement paste, minimizing agglomeration and improving workability. These characteristics make microencapsulated BPCMs more suitable for practical engineering applications compared to non-encapsulated PCMs.

Polyethylene glycol (PEG 6000) with an average molecular weight of approximately 6000 g/mol and a purity of ≥99% was used as the polymeric phase change material. The material was incorporated at a dosage of 10–15 wt% by mass of cement. PEG 6000 was selected due to its melting point of ~61 °C and latent heat capacity of ~170 J/g, which are well-suited for thermal regulation in building applications. Additionally, this grade exhibits good chemical compatibility with cementitious matrices, minimizing adverse interactions with hydration products. It is noted that PEGs with different molecular weights may display significantly different melting temperatures, latent heats, and dispersion behaviors. Therefore, the selected PEG 6000 was considered the most appropriate balance between phase change performance, stability, and practical applicability in cementitious composites.

Additionally, polyurethane-encapsulated BPCMs with high isocyanate ratios offer improved leakage resistance and thermal durability [92]. Overall, BPCMs show significant potential in improving the thermal regulation capabilities of cement paste. BPCMs may compromise mechanical strength. However, optimizing particle size, dosage, and encapsulation strategy enables a practical balance between structural integrity and thermal control [93].

In the context of increasing energy demands, cementitious materials incorporating BPCMs represent a promising solution for peak-load reduction via latent heat storage. These materials offer practical strategies for developing low-carbon, durable, and energy-efficient building envelopes [94,95]. However, the underlying mechanisms governing mechanical degradation remain insufficiently understood and are influenced by multiple interacting factors, including PCM type, dosage, particle size, encapsulation, and spatial distribution. Future studies should focus on the thermo-mechanical coupling mechanisms of different BPCMs under various climatic conditions and structural applications.

### 2.3. Machine Learning Model

To improve the generalization ability of the models, 80% of the data were allocated to the training set, while 20% were reserved for the validation set. This approach allows for the evaluation and comparative analysis of the influence of various material parameters on the performance of BPCM cement pastes.

#### 2.3.1. Support Vector Regression (SVR)

Support Vector Regression (SVR) is particularly effective for small-sample datasets and highly nonlinear regression tasks, owing to its reliance on the principle of structural risk minimization. The feature allows SVR to maintain strong generalization while mitigating overfitting, making it well-suited for modeling the thermophysical properties of complex multivariable systems such as cementitious composites containing BPCMs. However, the predictive accuracy of SVR is highly sensitive to the choice of kernel function and the setting of key hyperparameters. Suboptimal parameter configurations can result in underfitting or overfitting. To overcome this limitation, metaheuristic algorithms such as the Genetic Algorithm (GA), Particle Swarm Optimization (PSO), and Whale Optimization Algorithm (WOA) are employed to perform global hyperparameter tuning, avoiding local minima and improving model robustness [96]. n this study, Support Vector Regression (SVR) combined with metaheuristic optimization algorithms provides an efficient modeling strategy for systems characterized by limited data, nonlinear coupling, and unstable neural network convergence. This method offers a practical strategy toward accurate prediction of thermal behavior in BPCMs-modified cementitious materials [97].

#### 2.3.2. Random Forest (RF)

Random Forest (RF) is a bagging-based ensemble learning method composed of multiple decision trees. It is particularly advantageous in regression tasks involving limited datasets with complex input-output relationships [98]. RF models are robust to noise and outliers, make no assumptions about feature relationships, and output ranked feature importance metrics. These benefits support effective sensitivity analysis in multivariable systems. In the study, the optimized RF model demonstrated strong predictive performance across all three target properties: thermal conductivity, latent heat, and compressive strength [23,99]. Due to its robustness and adaptability, Random Forest is well-suited for data-driven analysis and prediction of thermal behavior in sustainable building materials, especially in small-sample datasets.

#### 2.3.3. Extreme Gradient Boosting (XGBoost)

XGBoost is an advanced implementation of Gradient Boosting Decision Trees (GBDT). It introduces several improvements over traditional GBDT models, including L1 and L2 regularization, optimized tree-splitting algorithms, built-in handling of missing values, and parallel computation [100]. These features yield high accuracy, efficiency, and robustness, even in high-dimensional, nonlinear scenarios [25]. XGBoost is well-suited for learning complex feature-response mappings without the need for explicit mathematical formulations. In this study, hyperparameter tuning was conducted via fold cross-validation and grid search [27]. Results show that XGBoost achieved low RMSE and high R^2^ in thermal property prediction, outperforming conventional regression models. Its ability makes XGBoost an efficient and accurate choice for modeling heat conduction and storage behavior in building materials.

#### 2.3.4. Categorical Boosting (CatBoost)

CatBoost is developed to handle categorical variables and complex, high-dimensional data structures, making it well-suited for material property prediction tasks where such challenges are common. Its core strengths include ordered target encoding, symmetric tree structures, and robust regularization strategies [101,102]. In this study, several input variables show strong categorical characteristics. CatBoost’s unique technique avoids target leakage while maintaining model stability in small datasets. Moreover, its symmetric tree structure ensures consistent decision paths across datasets, reducing variance and enhancing generalization. Experimental results indicate that CatBoost outperformed other models in predicting all three target variables, achieving high accuracy and stability [103].

### 2.4. Optimization Algorithms

#### 2.4.1. Genetic Algorithm (GA)

The accuracy and generalization capability of machine learning models such as SVR, RF, XGBoost, and CatBoost are highly sensitive to hyperparameter configurations, particularly in nonlinear, multivariate regression tasks [104,105]. Conventional methods such as grid or random search often fail to efficiently explore high-dimensional, non-convex parameter spaces, easily falling into local optima. A population-based metaheuristic inspired by natural selection and genetic evolution is introduced for global hyperparameter optimization [106]. The Genetic Algorithm encodes parameter sets as chromosomes and generates an initial population. It applies selection, crossover, and mutation operations guided by a fitness function, enabling global search and convergence toward optimal solutions [107,108].

#### 2.4.2. Particle Swarm Optimization (PSO)

Particle Swarm Optimization (PSO) is a widely adopted swarm intelligence algorithm that simulates the social behavior of bird flocks or fish schools. Each solution is represented as a particle, which updates its position based on both its own experience and the global best position identified by the swarm. This mechanism facilitates efficient global optimization in high-dimensional, nonlinear problem spaces. In this study, PSO is employed to optimize key hyperparameters of SVR, XGBoost, and CatBoost, including learning rate, subsample ratios, minimum split loss, and tree depth. PSO offers strong search capability, high computational efficiency, and easy parallelization. Compared with grid or random search, it achieves faster convergence in non-convex landscapes and is less prone to local optima.

#### 2.4.3. Whale Optimization Algorithm (WOA)

The Whale Optimization Algorithm (WOA) is a novel metaheuristic inspired by humpback whales’ bubble-net hunting strategy. WOA balances global and local search via two core mechanisms: spiral updating and encircling prey. Its structure and efficient convergence make it suitable for complex regression model tuning [109,110]. For SVR, WOA optimizes the penalty parameter, kernel type, and kernel width. For RF, it adjusts tree number, split criteria, and sampling ratio. For XGBoost and CatBoost, WOA searches over learning rate, tree depth, subsampling ratio, and regularization terms [111]. In this study, WOA optimizes model hyperparameters by minimizing prediction error as the fitness function.

#### 2.4.4. Grey Wolf Optimizer (GWO)

Grey Wolf Optimizer (GWO) is a method based on the social hierarchy and cooperative hunting behavior of grey wolves. It simulates the leadership roles of alpha, beta, delta, and omega wolves to navigate complex, high-dimensional solution spaces through exploration–exploitation balance [112]. In this study, GWO is used to optimize SVR, RF, and boosting model settings. GWO does not require gradient information and adapts well to multi-modal and discontinuous objective functions. Its hierarchical search procedure promotes robust convergence and effective escape from local minima [113,114]. models optimized by GWO are developed under the fitness criterion of minimizing model prediction error. They deliver strong regression performance and high generalization capacity, making them well-suited for nonlinear modeling of complex thermal behavior in cementitious composites.

#### 2.4.5. Firefly Algorithm (FFA)

The Firefly Algorithm (FFA) is a bio-inspired optimization technique based on the luminescent attraction behavior of fireflies. It is particularly suitable for solving nonlinear, multi-modal, and high-dimensional problems, making it effective for hyperparameter tuning in machine learning models [115,116]. In SVR, the Firefly Algorithm tunes the kernel function parameters and regularization terms. For Random Forest, it adjusts the number of trees, maximum depth, and sampling methods. In boosting models, it optimizes the learning rate, tree structure, and regularization parameters. Each candidate solution is treated as a firefly whose brightness corresponds to prediction accuracy. In the Firefly Algorithm, fireflies are attracted to brighter individuals based on their objective function values. This procedure enables global search while helping the algorithm avoid convergence to local optima [117].

### 2.5. Development of Predictive Models

In this study, four mainstream machine learning algorithms were employed to model and predict the performance of cementitious composites incorporating BPCMs. These algorithms include Support Vector Regression (SVR), Random Forest (RF), Extreme Gradient Boosting (XGBoost), and Categorical Boosting (CatBoost). A total of 100 experimental data samples were prepared, covering a wide range of parameter combinations including PCM dosage, water-to-cement ratio, curing age, and environmental conditions, to comprehensively capture multi-factor influences. The dataset was generated through laboratory experiments using ordinary Portland cement, fine and coarse aggregates, water, and BPCMs. Specimens (50 × 50 × 50 mm cubes) were cured at 20 ± 2 °C and ≥90% relative humidity. Compressive strength was tested according to the Chinese standard GB/T 50081-2019 [118] using a calibrated universal testing machine. Latent heat was measured with a differential scanning calorimeter (DSC, TA Instruments, New Castle, DE, USA), and thermal conductivity was determined using the transient plane source method with TPS (Hot Disk).

To promote generalization and predictive stability, the dataset was randomly divided into training and validation sets at an 8:2 ratio. A 10-fold cross-validation strategy was adopted for model evaluation to ensure robustness and minimize overfitting. To overcome the challenges of hyperparameter sensitivity and local optima, metaheuristic optimization algorithms were employed to fine-tune each model. The critical hyperparameters of the four ML models (SVR, RF, XGBoost, and CatBoost) were independently optimized using five metaheuristic algorithms, namely PSO, GA, WOA, GWO, and FFA. This procedure resulted in a total of 20 hybrid models. These optimization strategies significantly improved model accuracy and robustness by enabling global exploration of complex parameter spaces. For SVR, the penalty parameter C and kernel coefficient γ were optimized, controlling the balance between model flexibility and generalization. For RF, the number of trees (n_estimators) and maximum tree depth (max_depth) were tuned to regulate model complexity. For boosting-based models (XGBoost and CatBoost), learning rate, depth, and the number of estimators/iterations were adjusted to control convergence speed, model depth, and overall learning capacity. The population sizes and iteration numbers for the optimizers were chosen to balance computational efficiency and solution quality. Table 1 shows the search ranges and optimized hyperparameters.

**Figure 4:**
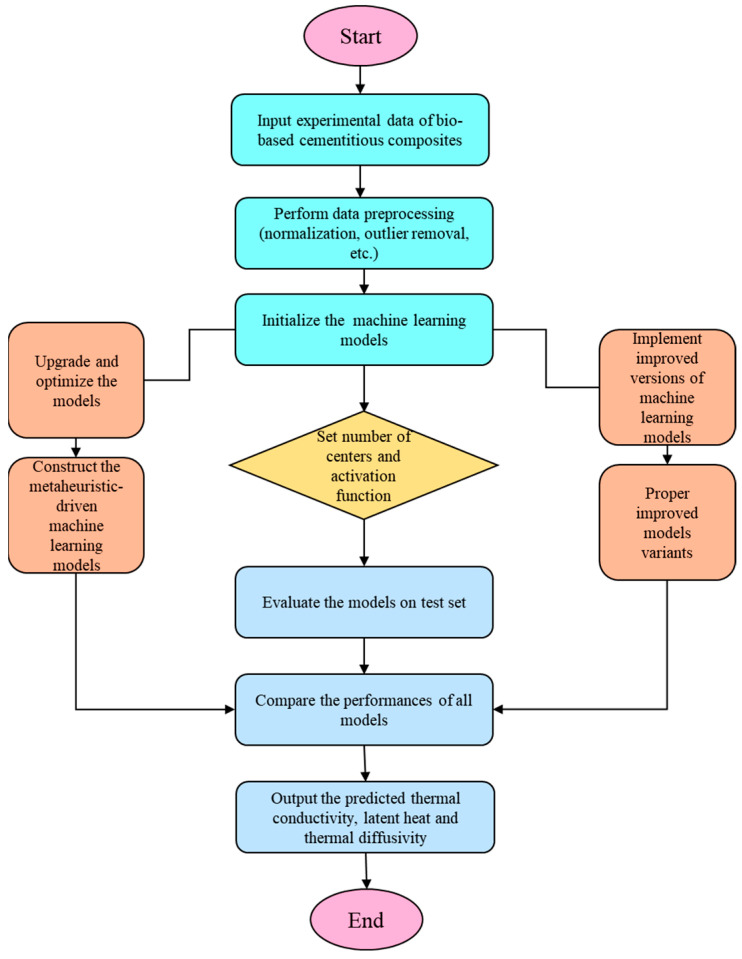
Modeling and optimization workflow for performance prediction of bio-based composite cementitious materials.

As illustrated in Figure 4, the overall modeling framework consists of four sequential stages: (1) Experimental data collection and feature construction; (2) Key factor analysis; (3) Model development and hyperparameter optimization; and (4) Model validation and performance assessment.

Each stage is logically interconnected, beginning with experimental data collection and progressing through feature analysis, model development, and optimization. The incorporation of metaheuristic algorithms into the workflow highlights the importance of intelligent hyperparameter tuning in improving model accuracy and generalizability. This structured pipeline ensures that both domain knowledge and computational efficiency are integrated to achieve reliable predictions for composite material behavior.

**Figure 5:**
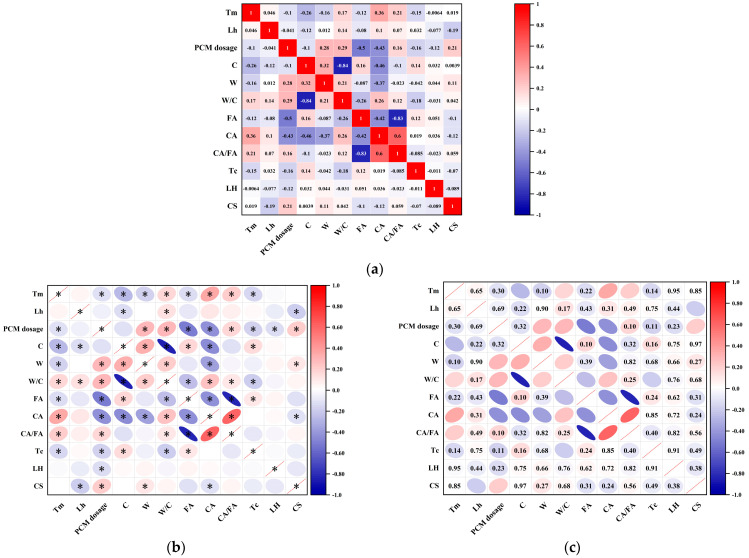
(**a**) Pearson correlation coefficient heatmap; (**b**) Significance annotation of correlations (significance level α = 0.1); (**c**) Combined map of correlation coefficients and significance levels.

Pearson correlation analysis was conducted to examine the linear associations among input variables, determine their statistical significance, and assess their potential impact on output targets [119]. Three types of graphical visualizations were used to present the results. Figure 5a presents a standard correlation heatmap. Figure 5b shows the statistical significance annotations for the variable correlations. Figure 5c combines the Pearson correlation coefficients with the corresponding *p*-values, using a significance threshold of α = 0.1.

As illustrated in Figure 5a, the Pearson correlation matrix reveals pairwise linear associations among the variables, with color gradients ranging from deep blue to deep red, and numerical values from −1 to +1 [120]. Most variable pairs show absolute correlation values |r| < 0.8, indicating a low risk of multicollinearity and satisfying the independence assumptions required for machine learning models. The strong negative correlation is observed between cement content (C) and coarse aggregate (CA), while a significant positive correlation is evident between water content (W) and water-to-cement ratio (W/C), reflecting inherent interdependencies in the concrete mix design parameters [121]. Figure 5b further annotates whether each correlation is statistically significant (*p* ≤ 0.1). Asterisks indicate non-significant relationships, while unmarked cells denote statistically significant correlations. Most strongly correlated variable pairs, such as C–CA and W–W/C, passed the significance test, confirming their statistical reliability. In addition, a significant negative correlation between compressive strength (CS) and PCM dosage was observed. This suggests that increasing PCM content may adversely affect the mechanical strength of the composite material [122]. Figure 5c shows correlation coefficients with *p*-values through ellipse-based visualization, where the orientation and shape of each ellipse reflect the direction and magnitude of the correlation [123]. According to the analysis, CS demonstrates moderate to strong linear relationships with several key variables, including cement content (r = 0.85), W/C (r = −0.84), thermal conductivity (Tc, r = −0.47), and latent heat (LH, r = −0.49), with most correlations statistically significant at *p* < 0.1.

The input variables show moderate correlation without severe multicollinearity. The majority of relationships meet the threshold for statistical significance [124,125]. This confirms a robust data foundation for the development of stable and interpretable machine learning models. Key variables such as cement content, W/C ratio, PCM dosage, and thermal properties (Tc and LH) reveal meaningful associations with compressive strength (CS), underscoring their importance for model construction [126].

**Figure 6:**
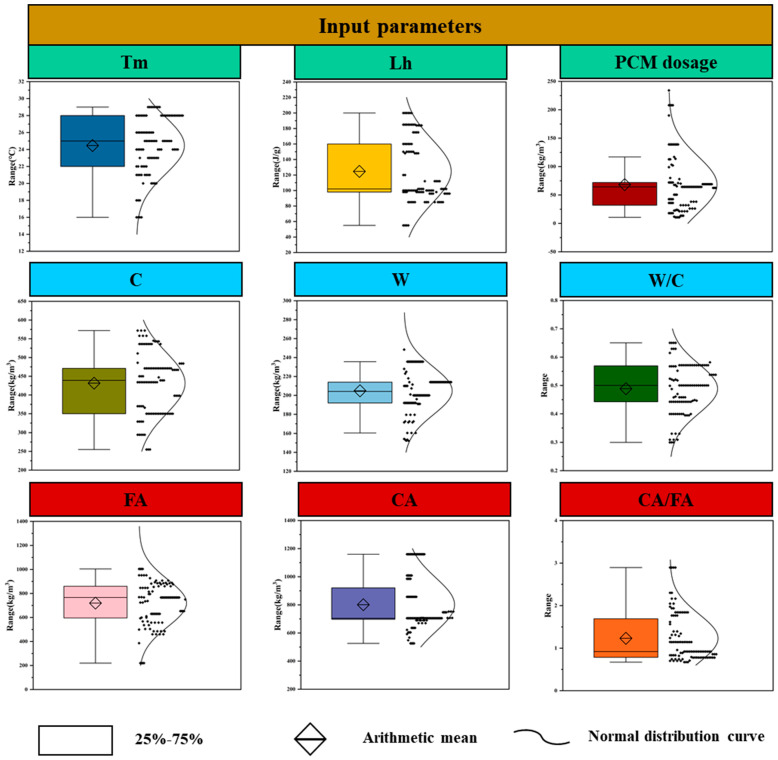
Boxplots of input variables (Tm, Lh, PCM dosage, C, W, W/C, FA, CA, and CA/FA).

**Figure 7:**
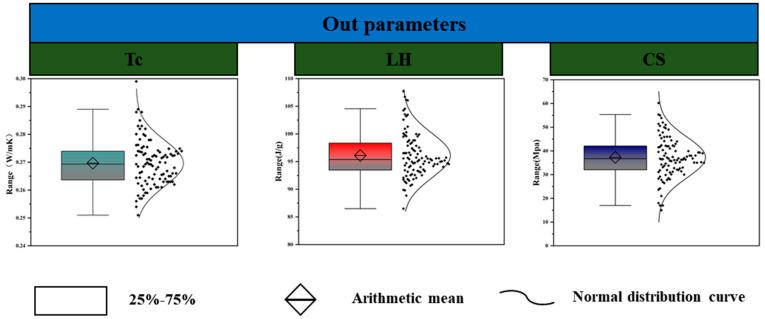
Boxplots of output variables (Tc, LH, and CS).

To evaluate the data structure and statistical properties of both input and output variables, the present study uses a combination of boxplot visualization and descriptive statistical metrics [127,128]. The results are illustrated in Figure 6 and Figure 7. Figure 6 presents the distribution patterns of nine input variables, including phase change temperature (Tm), latent heat (Lh), PCM dosage, cement content (C), water content (W), water-to-cement ratio (W/C), fine aggregate (FA), coarse aggregate (CA), and the CA-to-FA ratio (CA/FA). All features were standardized using z-score normalization. Preprocessing was performed exclusively within the training folds to avoid data leakage. No categorical variables were present in the dataset.

Most input variables display approximately symmetric distributions, with means close to their respective medians and no significant deviations in the boxplot. This indicates a well-centered dataset with a limited number of outliers. In terms of probability density, certain variables such as CA, FA, and W demonstrate slight concentration in the mid-to-high value ranges, suggesting sample design preferences [129]. Descriptive statistics further revealed that the standard deviations (SD) of coarse aggregate (CA) and fine aggregate (FA) were the highest among all variables. The respective values were approximately 172.3 kg/m^3^ for CA and 155.1 kg/m^3^ for FA, indicating substantial variation in aggregate design within the cementitious mixtures. In contrast, ratio-based variables such as W/C and CA/FA displayed lower SD values, indicating more consistent distributions. The W/C ratio in particular showed minimal variation, near-zero skewness, and high data quality due to standardized formulation practices [130,131]. Both PCM dosage and latent heat exhibited moderately skewed distributions, primarily concentrated in the 100–200 range. Their density curves were well-balanced on both sides, with no severe long-tail effects, indicating that the variables are suitable for direct modeling [90,132]. Skewness and kurtosis were computed to evaluate the symmetry and peakedness of the distributions. All variables present skewness values within the range of −2 to +2, and kurtosis within −10 to +10, which falls within the acceptable range suggested in the literature, indicating no significant data skewness or dispersion issues [92]. Figure 7 shows the distribution of output variables: thermal conductivity (Tc), latent heat (LH), and compressive strength (CS). Among them, CS displayed the widest range, spanning from 11.4 to 53.8 MPa, with a standard deviation of approximately 7.5 MPa. The distribution was nearly symmetric but slightly right-skewed (skewness = 0.38), indicating a broad coverage of strength grades. LH was primarily distributed in the 90–110 J/g range, with normal skewness and kurtosis, forming a symmetric probability density curve. Thermal conductivity (Tc) shows the narrowest range (approximately 0.25–0.29 W/m·K), reflecting limited variability.

The input and output variables exhibited favorable distributional characteristics, with no severe skewness or significant outliers. It provides a solid foundation for subsequent machine learning tasks such as feature selection, normalization, and model stability enhancement [133]. Furthermore, the descriptive statistical indicators, including the mean, standard deviation, skewness, and kurtosis, were analyzed in detail. These indicators support the conclusion that the data are suitable for regression-based prediction modeling [134,135].

## 3. Results and Discussion

### 3.1. Performance Prediction Based on Support Vector Regression and Optimized Hybrid Models

**Figure 8:**
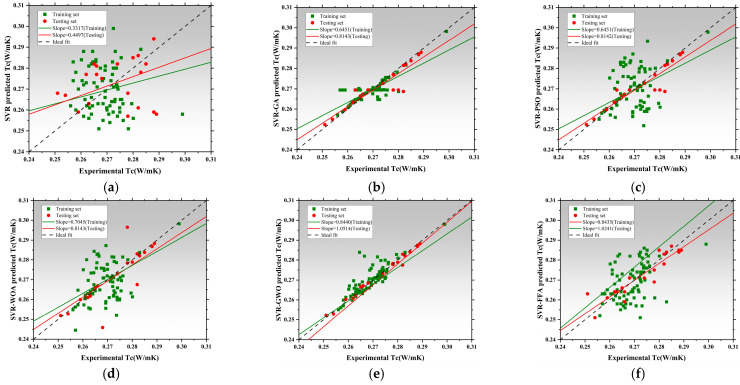
Regression graph for Tc. (**a**) SVR; (**b**) SVR-GA; (**c**) SVR-PSO; (**d**) SVR-WOA; (**e**) SVR-GWO; (**f**) SVR-FFA.

**Figure 9:**
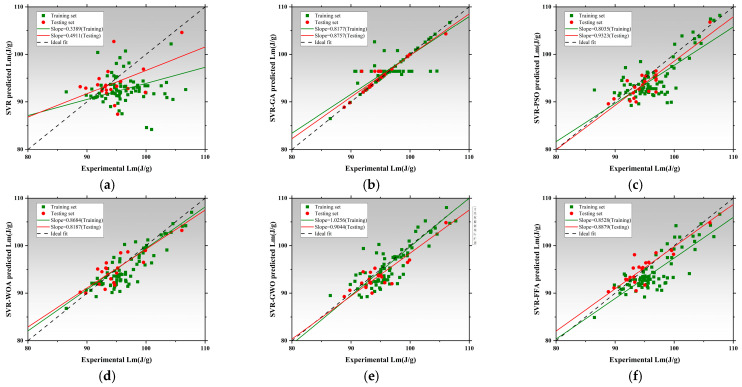
Regression graph for LH. (**a**) SVR; (**b**) SVR-GA; (**c**) SVR-PSO; (**d**) SVR-WOA; (**e**) SVR-GWO; (**f**) SVR-FFA.

**Figure 10:**
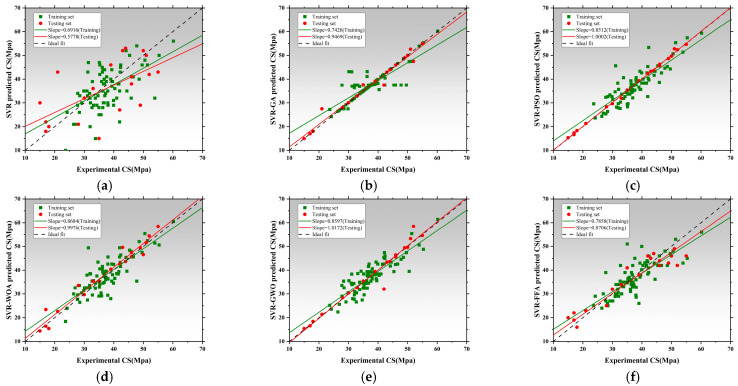
Regression graph for CS. (**a**) SVR; (**b**) SVR-GA; (**c**) SVR-PSO; (**d**) SVR-WOA; (**e**) SVR-GWO; (**f**) SVR-FFA.

Figure 8 presents the regression performance of six models in predicting thermal conductivity (Tc), including the SVR and five hybrid SVR models optimized by metaheuristic algorithms: SVR-GA, SVR-PSO, SVR-WOA, SVR-GWO, and SVR-FFA [136,137,138]. The x-axis represents measured values, while the y-axis corresponds to model-predicted values. The black dashed line denotes the ideal fit, while red and green lines represent regression trends for the training and testing datasets, respectively [139]. The slope of these lines reflects the consistency between predictions and actual values. The SVR exhibited poor regression performance, with training and testing slopes of 0.3317 and 0.4497, respectively. This indicates significant underfitting and limited capability in capturing the nonlinear features of thermal conductivity without parameter optimization [140]. SVR-GA and SVR-PSO achieved a testing slope of 0.8143, and SVR-PSO reached 0.8142, both achieving better model fit [141]. SVR-WOA further improved regression accuracy with training and testing slopes of 0.7045 and 0.8143, indicating improved fit and stronger generalization capability. SVR-GWO achieved the best overall performance, with regression slopes of 0.8440 for training and 1.0514 for testing, demonstrating exceptional ability to capture high-dimensional nonlinear patterns. SVR-FFA also resulted in stable performance with a testing slope of 1.0241, second only to SVR-GWO. All optimized SVR models outperformed the SVR, and SVR-GWO emerged as the best candidate for Tc prediction, improving the testing slope by 96.7% relative to the baseline [142]. Figure 9 illustrates model performance in predicting compressive strength. The SVR model performed inadequately, with slopes of 0.3389 and 0.4911, indicating an inability to capture complex mix–strength interactions. Optimization significantly improves performance [114]. SVR-GA achieved 0.8177 and 0.8757 for training and testing, respectively. SVR-PSO demonstrated near-perfect fit with a testing slope of 0.9323. SVR-WOA also showed high stability with slopes of 0.8684 and 0.8187. SVR-GWO achieved the highest performance, with slopes of 1.0256 and 0.9044, being the closest to the ideal fit among all models. SVR-FFA performed well with training and testing slopes of 0.8528 and 0.8879, respectively. SVR-GWO was the top-performing model in CS prediction [143], increasing testing slope by 76.2% over the base SVR and delivering high accuracy and generalization. Figure 10 presents the regression plots for latent heat prediction. The SVR again underperformed, with training and testing slopes of 0.6916 and 0.5778, reflecting typical underfitting. After optimization, SVR-GA improved testing slope to 0.9469. SVR-PSO further raised it to 0.8512, surpassing the 0.8 threshold for acceptable prediction [144,145]. SVR-WOA attained training and testing slopes of 0.8684 and 0.9976, respectively, demonstrating well-balanced predictive performance [146]. SVR-GWO achieved top performance, with slopes of 0.8597 and 1.0172. SVR-FFA also performed well with training and testing slopes of 0.7858 and 0.8706, respectively. All optimized models significantly outperformed base SVR in LH prediction, with SVR-GWO demonstrating the highest precision and robustness, improving the testing slope by 84%. The three sets of regression plots highlight differences in model capability across the three target variables [146,147]. The unoptimized SVR consistently exhibited underfitting, while models optimized with GWO, WOA, and FFA significantly improved both fitting accuracy and generalization. SVR-GWO achieved the highest or second-highest slope in all tasks [148], making it the most robust and accurate model overall.

**Figure 11:**
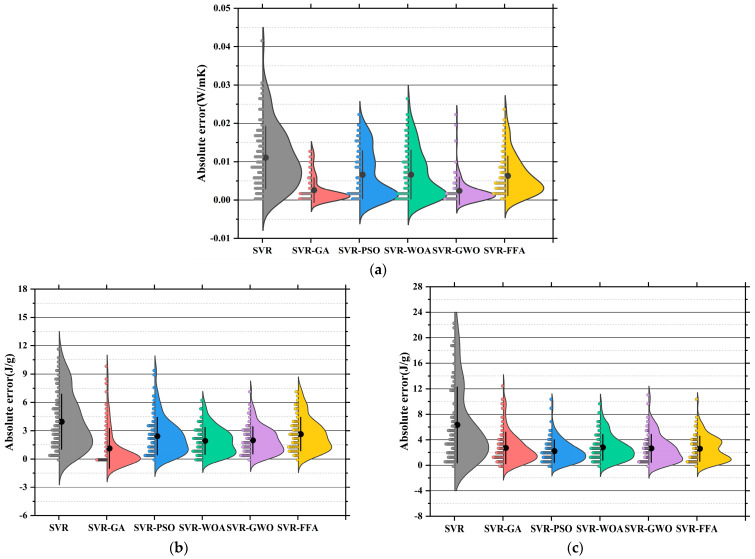
Comparison of error for SVR and its optimized models (**a**) Thermal conductivity (Tc); (**b**) Latent heat (LH); (**c**) Compressive strength (CS).

Figure 11a–c present the absolute error distribution (violin plots) for the models across the three prediction tasks. The base SVR model showed the widest error range and highest maximum error (~0.045 W/m·K), with mean and median far from zero. SVR-GA reduced the average error significantly, with most samples below 0.01 W/m·K. SVR-PSO further narrowed the error band. SVR-WOA and SVR-GWO exhibited the narrowest, most symmetrical distributions, with peak density near zero and average errors below 0.005 W/m·K. SVR-GWO displayed minimal outliers and near-zero error dispersion, indicating superior stability and precision. The SVR model showed highly dispersed errors, with a mean over 6 J/g and a maximum near 17 J/g. SVR-GA and SVR-PSO reduced mean errors to 3.5 J/g and 3.0 J/g, respectively. SVR-WOA and SVR-GWO further improved error control, with SVR-GWO achieving a mean of ~2.2 J/g and tightly clustered errors. SVR-FFA also performed well, though slightly less precise than GWO [149]. SVR has the largest error, with a mean exceeding 10 MPa and max nearing 25 MPa. SVR-GA and SVR-PSO improved accuracy significantly [150]. SVR-WOA and SVR-GWO provided the best overall control, with SVR-GWO showing a mean error near 3.1 MPa and a median near 2.5 MPa, indicating stable performance and low variability. SVR-FFA remained competitive for predictions below 3.8 MPa.

Across three tasks, SVR-GWO achieved the lowest errors, most concentrated distributions, and minimal outliers, confirming it as the most reliable and accurate model [151]. These results are consistent with the earlier regression slope analysis, further validating the effectiveness of GWO in hyperparameter tuning and global optimization [152].

### 3.2. Performance Prediction Based on Random Forest and Optimized Hybrid Models

**Figure 12:**
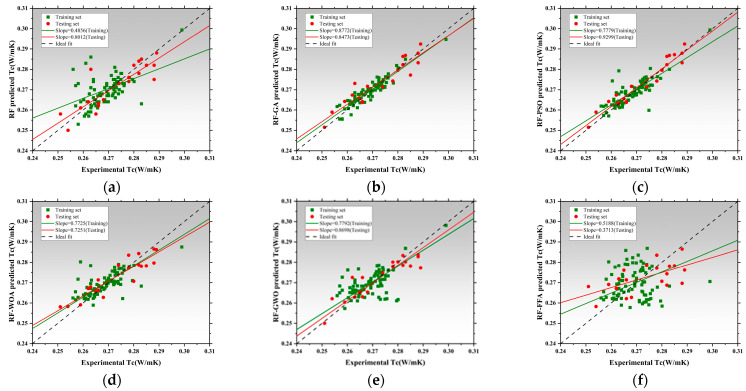
Regression graph for Tc. (**a**) RF; (**b**) RF-GA; (**c**) RF-PSO; (**d**) RF-WOA; (**e**) RF-GWO; (**f**) RF-FFA.

**Figure 13:**
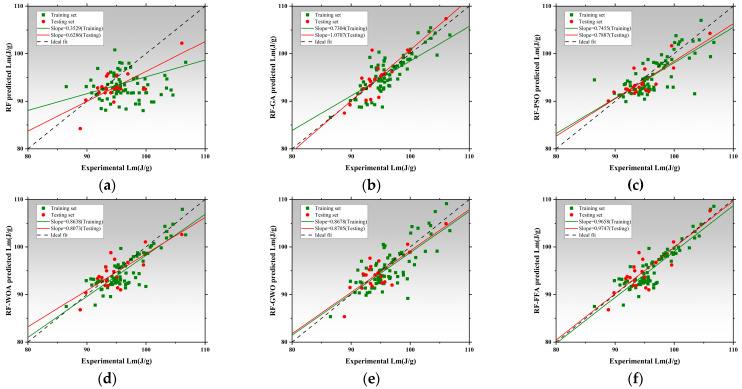
Regression graph for LH. (**a**) RF; (**b**) RF-GA; (**c**) RF-PSO; (**d**) RF-WOA; (**e**) RF-GWO; (**f**) RF-FFA.

**Figure 14:**
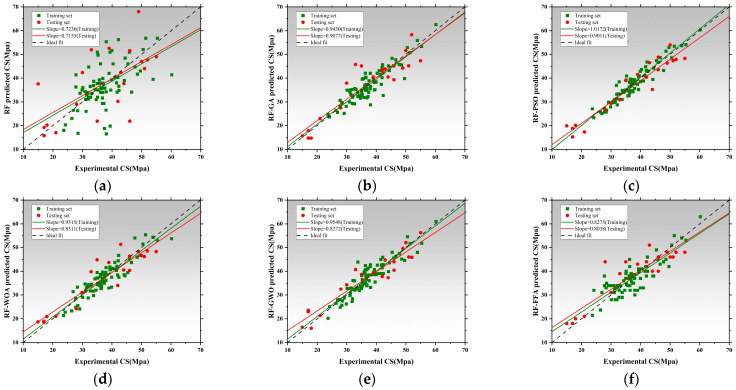
Regression graph for CS. (**a**) RF; (**b**) RF-GA; (**c**) RF-PSO; (**d**) RF-WOA; (**e**) RF-GWO; (**f**) RF-FFA.

Figure 12, Figure 13 and Figure 14 illustrate the training and testing regression fits for six models under each prediction task. The regression slope was used as a key indicator of prediction accuracy, with a value of 1 indicating perfect agreement between predicted and measured results [153,154]. Figure 12 shows the regression results for compressive strength. The RF model exhibited relatively low regression slopes of 0.4856 and 0.8012, indicating limited fitting capacity and poor generalization under default hyperparameters. After applying genetic algorithm optimization, the RF-GA model significantly improved, with slopes increasing to 0.8772 and 0.8473, suggesting strong fitting and no signs of overfitting [154]. The RF-PSO model achieved the best performance, with training and testing slopes of 0.7779 and 0.9299, respectively, representing near-perfect fit and outstanding generalization [155,156]. Both RF-WOA and RF-GWO also achieved high accuracy, with training slopes of 0.7725 and 0.7251, and testing slopes of 0.7792 and 0.8698. The small slope differences (<0.03) indicate strong model stability. Its slightly lower performance compared to other optimized models may be attributed to mild underfitting. Overall, RF-PSO achieved the best accuracy in CS modeling, followed by RF-GA and RF-GWO [157]. Figure 13 presents regression results for latent heat. The RF model underperformed, with training and testing slopes of 0.3529 and 0.6286. Optimized models showed clear improvements. RF-GA achieved a training slope of 0.7304 and a testing slope of 1.0707. RF-PSO reached 0.7455 and 0.7887 for training and testing, respectively. Both are close to the 0.8 threshold, indicating enhanced nonlinear learning ability [158]. The RF-WOA model further improved predictive accuracy, achieving a testing slope of 0.8638. While the RF-GWO model showed the best results with training and testing slopes of 0.8678 and 0.8705, respectively. The small slope difference of 0.0212 highlights the model’s high robustness and low risk of overfitting. RF-FFA also performed well, with training and testing slopes of 0.9658 and 0.9747, closely trailing RF-GWO. Overall, RF-GWO showed the strongest generalization and precision in LH prediction, with RF-FFA and RF-WOA as strong alternatives. Figure 14 shows regression results for thermal conductivity (Tc), a task that involves small-range, low-variance data distributions, making it sensitive to model granularity and error [159]. The RF model achieved poor regression slopes of 0.7236 and 0.7155, indicating poor learning of input–output mapping and weak generalization. The RF-GA model showed moderate improvement, with slopes of 0.9430 and 0.9077 for training and testing, respectively [160]. Among all models, RF-GWO achieved the highest performance, with a training slope of 0.9548 and a testing slope of 0.8272, confirming the effectiveness of GWO for hyperparameter tuning [161]. RF-FFA showed weaker generalization, with a training slope of 0.8275.

The RF model showed the weakest performance across all tasks, with significant bias and poor stability. All optimization algorithms improved prediction accuracy and generalization to varying extents. RF-GWO consistently achieved the highest or second-highest slopes across all tasks, especially in LH and Tc predictions, making it the most balanced and robust model [162].

**Figure 15:**
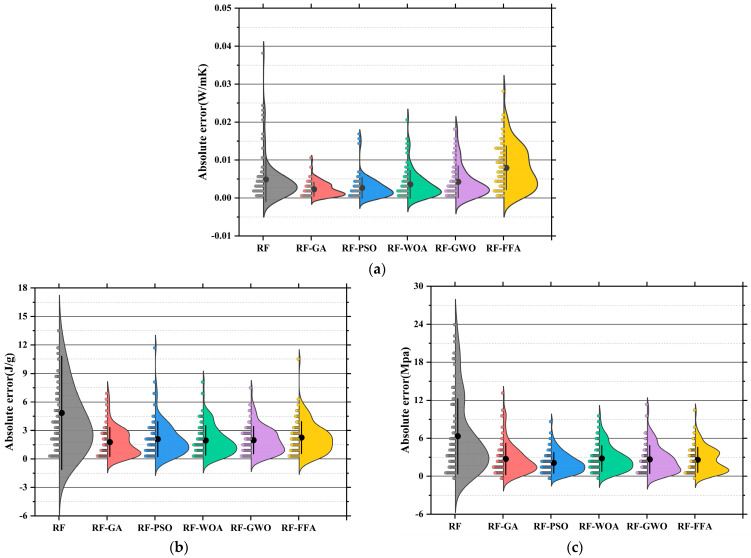
Comparison of error distributions for RF and its optimized models (**a**) Thermal conductivity (Tc); (**b**) Latent heat (LH); (**c**) Compressive strength (CS).

Figure 15a displays the absolute error distributions for the Tc prediction. The RF model showed the widest error range (>0.045 W/m·K), with a right-skewed density curve and long tails, indicating high variance and instability [163]. After optimization, both RF-GA and RF-PSO significantly reduced prediction errors, with most samples falling within ±0.015 W/m·K. RF-WOA and RF-GWO achieved better performance [164], with highly concentrated and nearly symmetric error distributions. In particular, RF-GWO showed minimal outliers and a dominant error range within ±0.01 W/m·K, reflecting strong precision and generalization. While RF-FFA remained below 0.03 W/m·K, its distribution was slightly skewed with heavier tails, suggesting lower stability than RF-GWO. Hence, RF-GWO was the most accurate and stable model for Tc prediction. Figure 15b illustrates absolute errors for LH prediction. The RF model had the widest spread, with errors up to ±17 J/g and a low-density, wide main region [165]. Optimized models significantly narrowed the error range. RF-GA and RF-PSO constrained most errors within ±6 J/g, confirming enhanced learning ability. RF-WOA and RF-GWO showed even tighter distributions. Particularly, RF-GWO had a high-density peak concentrated within ±2 J/g and minimal tail extension, indicating excellent robustness and convergence. RF-FFA also performed well but was slightly more dispersed. Overall, RF-GWO demonstrated the best error control and stability in LH prediction [166,167]. Figure 15c shows the error distribution for CS prediction. The RF model exhibited the widest error spread, with deviations reaching up to ±17 J/g and a low-density, broadly distributed main region. Optimized models crucially reduced errors. RF-GA and RF-PSO lowered most errors to within ±8 MPa, though some instability remained. In contrast, RF-WOA and RF-GWO achieved superior error convergence. RF-GWO had the most compact distribution, with the main density centered within ±3 MPa and almost no long tails, highlighting its high accuracy in modeling structural strength. RF-FFA was slightly less precise than GWO but still significantly outperformed the baseline RF [168].

In conclusion, RF-GWO achieved highly accurate predictions and tightly clustered outputs, confirming its robustness and high predictive accuracy across all three tasks [169]. Its superior performance validates GWO’s effectiveness in optimizing high-dimensional, nonlinear predictive models.

### 3.3. Performance Prediction Based on Extreme Gradient Boosting and Optimized Hybrid Models

**Figure 16:**
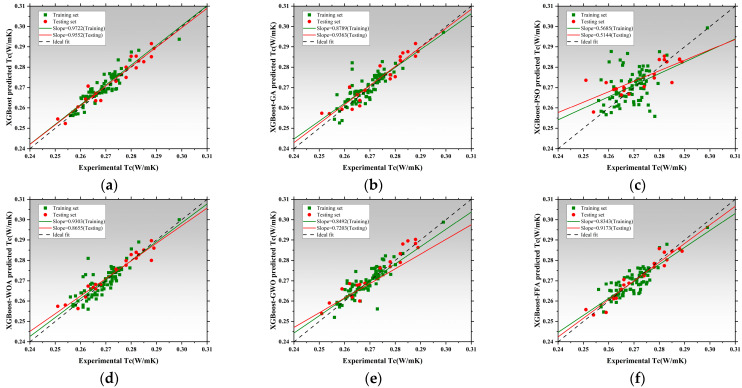
Regression graph for Tc. (**a**) XGBoost; (**b**) XGBoost-GA; (**c**) XGBoost-PSO; (**d**) XGBoost-WOA; (**e**) XGBoost-GWO; (**f**) XGBoost-FFA.

**Figure 17:**
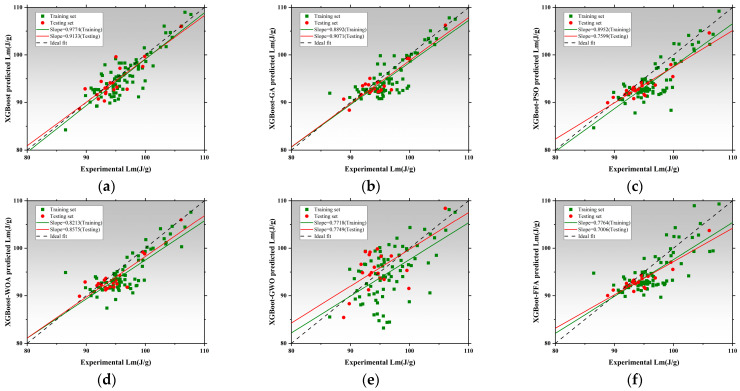
Regression graph for LH. (**a**) XGBoost; (**b**) XGBoost-GA; (**c**) XGBoost-PSO; (**d**) XGBoost-WOA; (**e**) XGBoost-GWO; (**f**) XGBoost-FFA.

**Figure 18:**
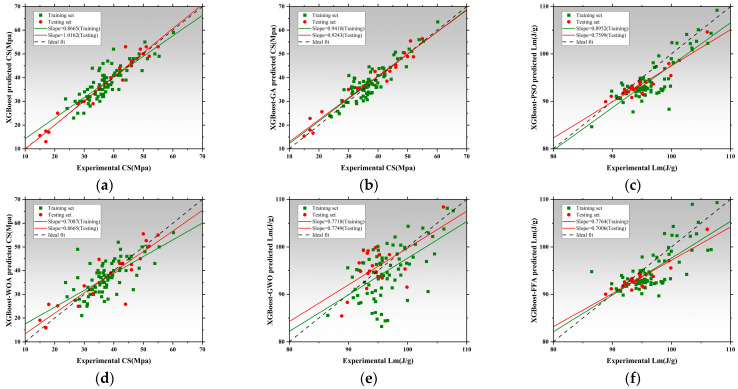
Regression graph for CS. (**a**) XGBoost; (**b**) XGBoost-GA; (**c**) XGBoost-PSO; (**d**) XGBoost-WOA; (**e**) XGBoost-GWO; (**f**) XGBoost-FFA.

Figure 16 presents the regression analysis of thermal conductivity prediction using the XGBoost model and its five optimized models [170]. XGBoost achieved regression slopes of 0.9722 and 0.9552 for training and testing, respectively, demonstrating its strong ability to learn both linear and nonlinear data structures. The XGBoost-GA slightly underperformed, with testing slope decreasing to 0.9363, suggesting limited improvement via a genetic algorithm in this task [171,172]. The XGBoost-PSO achieved a training slope of 0.5685 but drops to 0.5144 on the test set, indicating overfitting. In contrast, XGBoost-WOA and XGBoost-GWO both maintained high slopes above 0.90. XGBoost-WOA achieves a testing slope of 0.8655, showcasing strong accuracy and generalization [173]. XGBoost-GWO performs slightly below, with a testing slope of 0.7203. XGBoost-FFA exhibits regression slopes of 0.8343 and 0.9173, slightly inferior to the WOA model. Overall, XGBoost-WOA demonstrates the best balance between precision, robustness, and generalizability in Tc prediction. Figure 17 illustrates model performance in latent heat (LH) prediction. XGBoost achieved slopes of 0.9774 and 0.9133, reflecting high accuracy but also some sensitivity to nonlinear data deviations from the ideal fit line [174,175]. The XGBoost-PSO model significantly boosted predictive stability, with slopes of 0.8952 and 0.7599, reflecting improved fit. The XGBoost-WOA model performed best overall, achieving 0.8213 and 0.8575, confirming WOA’s effectiveness in modeling complex characteristics [176]. However, XGBoost-GWO showed signs of overfitting, with training and testing slopes of 0.7718 and 0.7749, respectively. XGBoost-FFA showed the weakest testing slope, indicating inadequate generalization and possible underfitting. Therefore, XGBoost-WOA stands out as the optimal model for LH prediction, offering both high accuracy and model robustness. Figure 18 presents the regression performance for predicting compressive strength. The baseline model shows high training and testing slopes, with the latter exceeding unity, implying slight overestimation in high-strength ranges. The XGBoost-GA improves generalization, with training and testing slopes of 0.9410 and 0.9243. XGBoost-PSO exhibits balanced performance, indicating strong robustness. XGBoost-WOA achieves 0.9414 but lower performance on testing, suggesting reduced generalization. XGBoost-GWO attains 0.9072 and 0.9175, within the accepted threshold and thus still viable for nonlinear CS prediction [177]. The best performance is delivered by XGBoost-FFA, which achieves 0.9403 and 1.0422, closely matching the ideal regression line, and demonstrating excellent predictive consistency on unseen data. All XGBoost models perform well for CS prediction, with XGBoost-PSO and XGBoost-FFA offering the most stable and accurate results, suitable for engineering applications [178].

**Figure 19:**
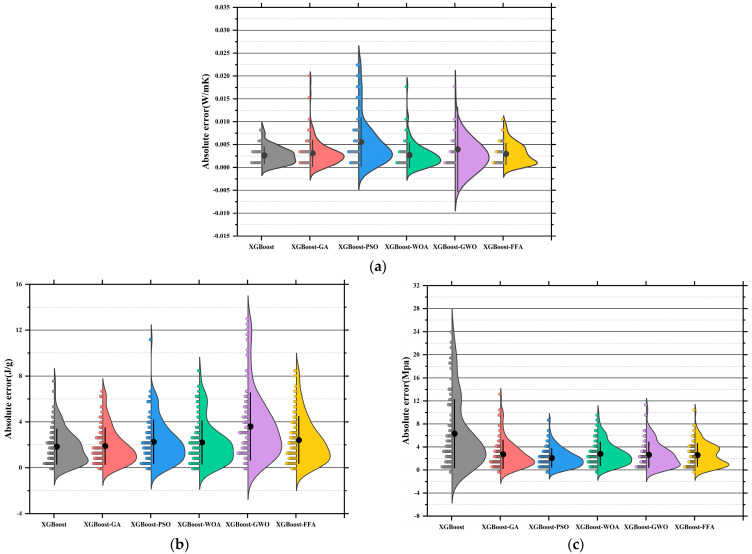
Comparison of error distributions for XGBoost and its optimized models (**a**) Thermal conductivity (Tc); (**b**) Latent heat (LH); (**c**) Compressive strength (CS).

Figure 19a presents the absolute error distributions for Tc prediction. The baseline model showed skewed error behavior, with maximum deviations nearing 0.025 W/m·K. Optimization significantly reduces error ranges. XGBoost-FFA and XGBoost-PSO demonstrated the most concentrated error distributions, with maximum errors constrained within 0.015 W/m·K and 0.018 W/m·K, respectively. Both models show strong reliability. XGBoost-GA and XGBoost-WOA also show excellent error control, with median errors close to zero and most predictions within ±0.01 W/m·K. While XGBoost-GWO showed some tail extension at the boundaries, its distribution remains symmetrical and centered, indicating high noise resistance. Overall, XGBoost-FFA provided the most accurate and stable performance in Tc prediction [179]. Figure 19b shows LH prediction errors. While the baseline model performed adequately, it presents outliers with maximum errors exceeding 12 J/g. Optimized models significantly compress error ranges. XGBoost-GA and XGBoost-PSO maintained most prediction errors within ±3 J/g, with near-Gaussian distributions. XGBoost-WOA and XGBoost-GWO exhibit slightly wider but still well-centered distributions within ±4 J/g, indicating strong resilience to parameter variation. XGBoost-FFA outperformed all others with the smallest average error and a highly concentrated distribution in the 0–2 J/g range [180]. Thus, XGBoost-GA and XGBoost-FFA achieved the best balance between accuracy and robustness in LH modeling [181]. Figure 19c shows error distributions for CS prediction. The baseline model exhibits considerable variability, with errors approaching 28 MPa, suggesting poor fit for high-strength samples. All optimized models reduce this significantly. XGBoost-GA and XGBoost-PSO reduced the maximum errors to below 10 MPa., with most errors in the 0–5 MPa range. XGBoost-WOA and XGBoost-GWO further refined model fit in medium-to-high strength ranges, showing tighter distributions and lower peak deviations. XGBoost-FFA deliverd the most symmetric distribution and lowest median error [182], maintaining strong generalization even under nonlinear and heterogeneous data conditions. Overall, optimized XGBoost models outperformed with FFA and PSO models offering the greatest potential for practical engineering deployment [183,184].

### 3.4. Performance Prediction Based on Categorical Boosting and Optimized Hybrid Models

**Figure 20:**
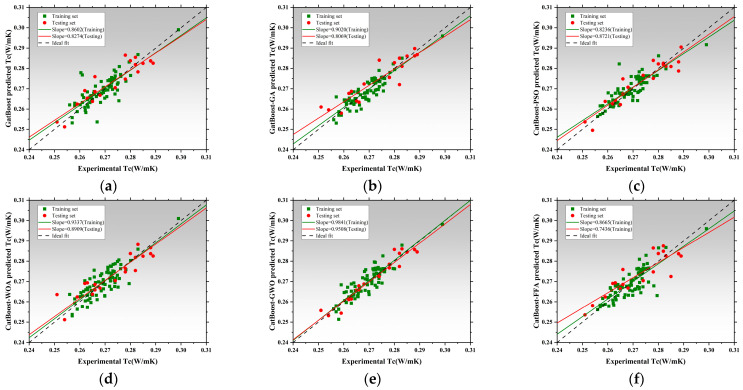
Regression graph for Tc. (**a**) CatBoost; (**b**) CatBoost-GA; (**c**) CatBoost-PSO; (**d**) CatBoost-WOA; (**e**) CatBoost-GWO; (**f**) CatBoost-FFA.

**Figure 21:**
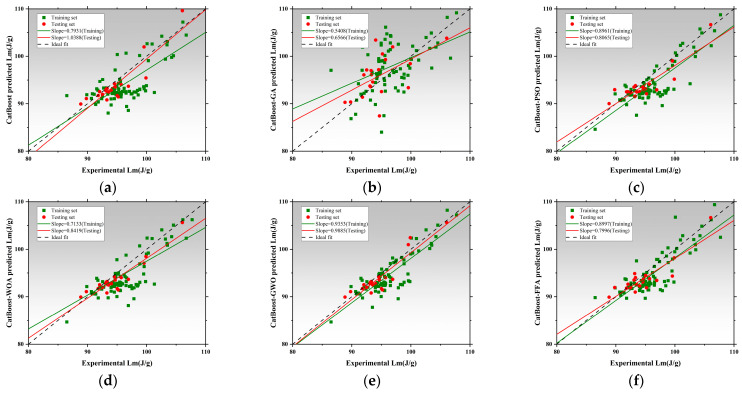
Regression graph for LH. (**a**) CatBoost; (**b**) CatBoost-GA; (**c**) CatBoost-PSO; (**d**) CatBoost-WOA; (**e**) CatBoost-GWO; (**f**) CatBoost-FFA.

**Figure 22:**
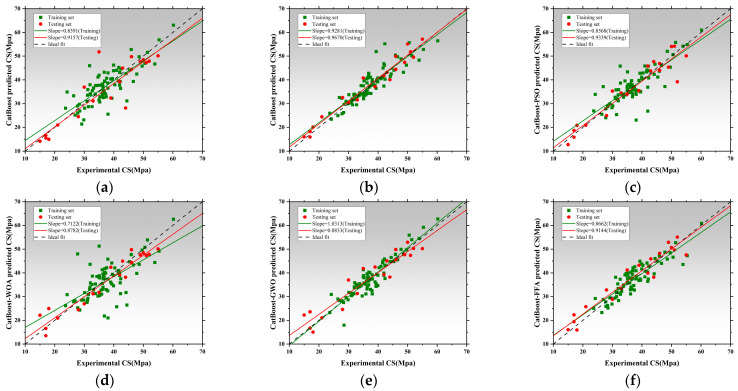
Regression graph for CS. (**a**) CatBoost; (**b**) CatBoost-GA; (**c**) CatBoost-PSO; (**d**) CatBoost-WOA; (**e**) CatBoost-GWO; (**f**) CatBoost-FFA.

**Figure 23:**
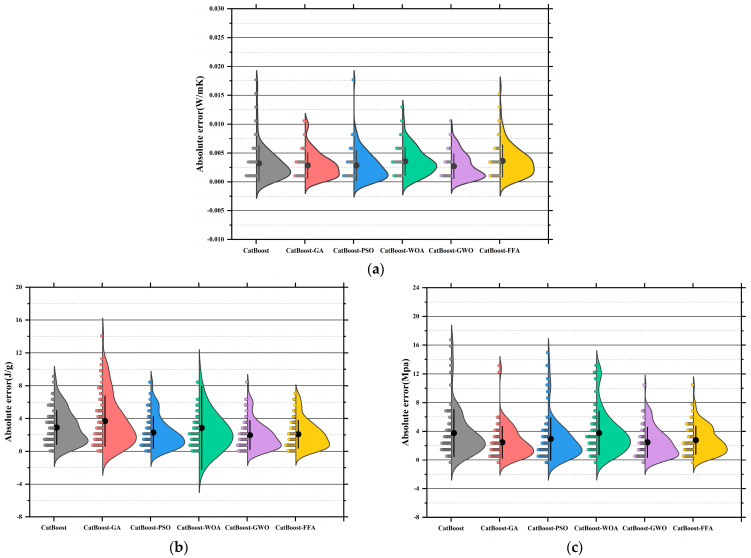
Comparison of error distributions for CatBoost and its optimized models (**a**) Thermal conductivity (Tc); (**b**) Latent heat (LH); (**c**) Compressive strength (CS).

Figure 20 illustrates the regression performance of the CatBoost model and its optimized models in predicting thermal conductivity. The CatBoost model showed regression slopes of 0.8602 and 0.8274, indicating limited predictive capability and relatively large prediction errors. After genetic algorithm optimization, the slopes improved to 0.9020 and 0.8069, respectively, indicating the improvement in both model fitting and generalization capability [185,186]. While the CatBoost-PSO model improved the training slope to 0.8236, the testing slope declined slightly to 0.8721. This implies better fitting on the training data but reduced ability to generalize. The CatBoost-WOA, CatBoost-GWO, and CatBoost-FFA models all showed significant improvements. Among them, CatBoost-WOA reached a testing slope of 0.8909, CatBoost-GWO achieved 0.9508, and CatBoost-FFA followed closely with 0.7436. These results confirm the efficacy of metaheuristic optimization [187,188]. In particular, CatBoost-GWO showed high slope consistency between training and testing, with only a 0.014 difference, indicating strong generalization. Overall, CatBoost-GWO achieved the best performance in predicting Tc [189,190]. Figure 21 displays the regression analysis for latent heat prediction. The significant discrepancy suggests underfitting during training and possible reliance on data noise [191]. Optimization with GA markedly improved performance with a minimal slope difference, indicating excellent fitting and model stability. Similarly, CatBoost-PSO achieved 0.8236 and 0.8721, though slightly overfitting the training data. The best performing models were CatBoost-GWO and CatBoost-FFA, both approaching the ideal regression line [192,193]. CatBoost-FFA achieved the minimal slope difference of 0.0008. With a testing slope of 0.9996, CatBoost-FFA was the top performer in LH prediction, followed closely by CatBoost-GWO. While the baseline and WOA-optimized models performed less effectively. Figure 22 shows the regression results for compressive strength prediction. The CatBoost model achieved slopes of 0.8391 and 0.9157, reflecting moderate accuracy. However, its departure from the ideal fit points to underfitting. After optimization, CatBoost-GA demonstrated significant improvement, achieving slopes of 0.9281 for training and 0.9670 for testing. The small slope gap reflects strong fitting performance and high model stability. CatBoost-PSO achieved 0.8568 and 0.9339, showing excellent fitting though with a mild overfitting tendency [194]. In contrast, CatBoost-WOA performed weakly, with 0.7122 and 0.8782, the lowest among all models, suggesting poor generalization and insufficient capture of input-output relationships [195]. CatBoost-GA performed best in CS prediction, followed closely by CatBoost-GWO and CatBoost-FFA, while CatBoost-WOA lagged behind, possibly due to overfitting or inadequate feature learning [196]. Figure 23 presents violin plots of absolute error distributions for Tc, LH, and CS predictions using CatBoost and its optimized models. The error offers an intuitive comparison of each model’s predictive precision and stability. In the thermal conductivity prediction, the CatBoost model exhibited right-skewed error distribution, with maximum errors exceeding 0.025 W/m·K, while the minimum was near zero. In contrast, CatBoost-FFA and CatBoost-GWO exhibited tighter and more centered distributions, with peak density around ±0.005 W/m·K. CatBoost-FFA showed the most concentrated density, confirming its high accuracy and robustness [197]. CatBoost-GA also improved prediction, with a median error of 0.008 W/m·K, compared to 0.014 W/m·K. In latent heat prediction, the baseline model reached a maximum error of nearly 16 J/g. However, CatBoost-FFA and CatBoost-WOA reduced this to within ±6 J/g. CatBoost-PSO also performed well, with 70% of errors falling within ±4 J/g, demonstrating good stability. CatBoost-GA had a wider error spread but still improved significantly over the baseline, with a median error around 2.5 J/g, compared to 6 J/g in the baseline. For compressive strength, differences were more pronounced [198]. The baseline model had maximum errors exceeding 20 MPa, with a wide error range and significant outliers; the median absolute error was approximately 7.2 MPa. Optimized models CatBoost-GWO and CatBoost-FFA again stood out, with concentrated error distributions, high peak densities, and over 85% of predictions within ±6 MPa. CatBoost-FFA achieved the lowest median error at ~2.5 MPa, nearly half that of the baseline, indicating strong generalization and superior performance under high nonlinearity and variability [143,199].

All optimized models improved accuracy and robustness across all three prediction tasks. Among all models, CatBoost-FFA exhibited the most balanced and superior performance. It attained the lowest average absolute errors for Tc (0.0035 W/m·K), LH (2.2 J/g), and CS (2.5 MPa), indicating its strong generalization across all targets [200]. These findings confirm its high potential for accurate multi-performance prediction in cementitious composites.

### 3.5. Comparative Evaluation of Model Accuracy

In this study, the predictive performance of each model was comprehensively evaluated using three statistical metrics [201]: Root Mean Square Error (RMSE), Mean Absolute Error (MAE), and the Coefficient of Determination (R^2^). The mathematical formulations of these evaluation indices are presented in Equations (1)–(3). RMSE quantifies the overall magnitude of prediction errors and is more sensitive to large deviations, making it suitable for assessing the global performance of regression models. MAE measures the average absolute difference between predicted and observed values, offering a direct and interpretable measure of model accuracy. The R^2^ index, on the other hand, reflects the proportion of variance in the observed data explained by the model; values closer to 1 indicate a higher level of goodness-of-fit and stronger predictive capability. Together, these three indicators provide a robust framework for evaluating the models’ precision, bias, and generalization ability under varying performance prediction tasks.

(1) $$RMSE=\sqrt{\frac{\sum_{i=1}^{n}(x_{i}-{\hat{x}}_{i}{)}^{2}}{n}}$$

(2) $$MAE=\frac{\sum_{i=1}^{n}\left|x_{i}-{\hat{x}}_{i}\right|}{n}$$

(3) $$R^{2}=1-\frac{\sum_{i=1}^{n}{\left({\hat{x}}_{i}-x_{i}\right)}^{2}}{\sum_{i=1}^{n}{\left(x_{i}-\bar{x}\right)}^{2}}$$

where $x_{i}$ is the i-th measured (experimental) value, ${\hat{x}}_{i}$ is the i-th predicted value, and n represents the total number of data points.

**Figure 24:**
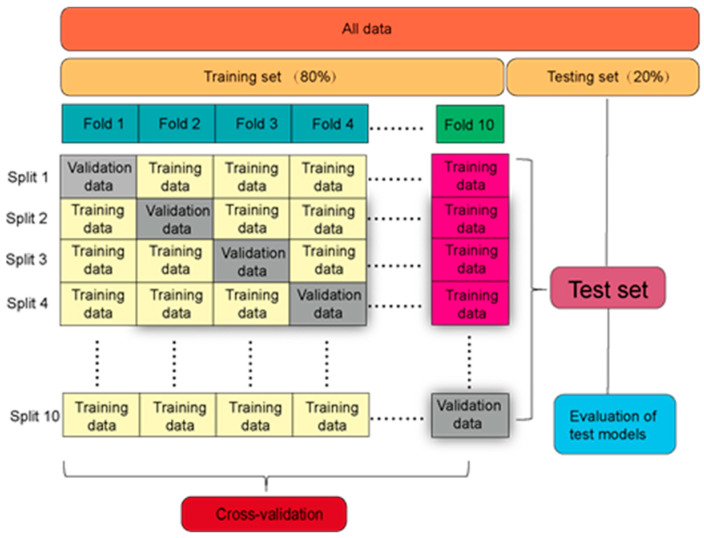
Training–testing data splitting and validation flowchart based on K-fold cross-validation.

To ensure the generalization capability and predictive stability of the developed machine learning models, the data partitioning and model validation process adopted in this study is illustrated in Figure 24. The entire dataset was first divided into a training set (80%) and a testing set (20%). The training set was used for model training and hyperparameter optimization, while the testing set was reserved exclusively for independent performance evaluation, ensuring objectivity and reliability of the final results [202,203].

During the training process, a 10-fold cross-validation strategy was applied to the training set [204]. Specifically, the training data was randomly partitioned into ten equal subsets. In each iteration, one subset was used as the validation set and the remaining nine were used for model training [205,206]. This process was repeated ten times, with each subset being used exactly once for validation. Hyperparameter optimization and model selection were conducted using 10-fold cross-validation within the training set, with all preprocessing restricted to avoid data leakage. Performance metrics were reported as mean CV fold values and separately for the test set.

After cross-validation, the optimal hyperparameter configuration was determined, and the final model was then evaluated on the previously unseen testing set. This procedure provides a comprehensive assessment of the model’s ability to generalize to new data [207,208]. Overall, this strategy effectively mitigates the risk of overfitting and significantly improves the accuracy and credibility of model evaluation. It is widely regarded as a standard and rigorous approach in contemporary machine learning research.

**Figure 25:**
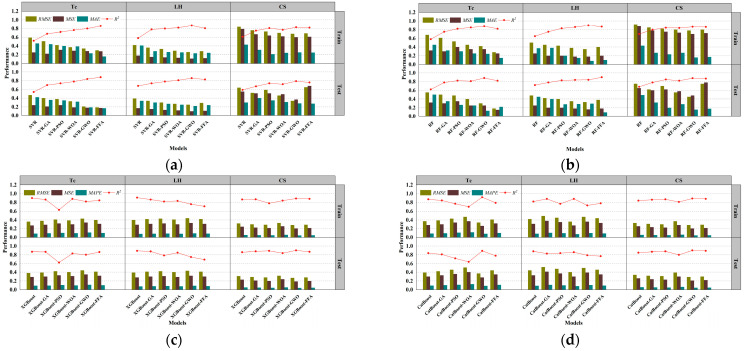
Comparison of Predictive Models on Tc, LH, and CS. (**a**) SVR; (**b**) RF; (**c**) XGBoost; (**d**) CatBoost.

Figure 25 presents the performance comparison of four machine learning models and their combinations with five metaheuristic optimization algorithms, across three key prediction targets: thermal conductivity (Tc), latent heat (LH), and compressive strength (CS). Each model was evaluated on both the training and testing datasets using four commonly adopted metrics: Root Mean Square Error (RMSE), Mean Absolute Error (MAE), and Coefficient of Determination (R^2^) [209]. The SVR model, as a classical regression method, exhibited suboptimal performance in its baseline configuration, particularly in predicting CS, where the testing R^2^ was only 0.748 with an RMSE of 6.23 MPa—highlighting its limited capability in capturing complex nonlinear interactions. However, its performance improved significantly after optimization via swarm intelligence algorithms [210]. For instance, the SVR-WOA model achieved an R^2^ of 0.912 in CS prediction, with MAE reduced to 3.64 MPa, indicating enhanced generalization ability. Similarly, the SVR-FFA model demonstrated the best performance in LH prediction (R^2^ = 0.893, RMSE = 2.15 J/g), with its prediction errors concentrated within a low-value range [211].

The RF model showed inherently stronger stability in nonlinear tasks. Without optimization, it achieved an R^2^ of 0.843 for CS prediction, outperforming SVR. The RF-WOA model further improved the performance, achieving an R^2^ value of 0.928. T The RF-FFA model attained the best performance in Tc prediction, with an RMSE of 0.0078 W/m·K and an R^2^ value of 0.886, demonstrating strong accuracy in thermal performance modeling [212]. In latent heat prediction, RF-PSO and RF-GWO both maintained R^2^ values above 0.87 with low error variance, reflecting high prediction robustness. XGBoost models exhibited superior predictive accuracy across all indicators. The XGBoost model already achieved a high testing R^2^ of 0.911 for CS prediction. After optimization, the XGBoost-WOA model achieved an R^2^ of 0.932 and an RMSE of 1.97 J/g in LH prediction, indicating strong adaptability to nonlinear features and complex data distributions [213,214]. With an RMSE of 0.0064 W/m·K and an R^2^ of 0.905, the XGBoost-GWO model demonstrated strong predictive performance., ranking among the best-performing models. CatBoost, a gradient boosting framework based on symmetric tree structures, delivered the most outstanding performance across the board [215]. The CatBoost model achieved an R^2^ of 0.936 for CS prediction. Its optimized version, CatBoost-WOA, yielded an R^2^ of 0.955 and an MAE of 1.84 J/g for LH prediction, representing the most accurate results among all compared models. For Tc prediction, the CatBoost-FFA model demonstrated strong performance, with an RMSE of 0.0057 W/m·K, and an R^2^ of 0.927. These results indicate that it outperformed other models in both generalization capability and fine-grained fitting accuracy [216]. CatBoost-WOA and XGBoost-WOA emerged as the top-performing ensemble models in thermal and mechanical property predictions, respectively [217].

**Figure 26:**
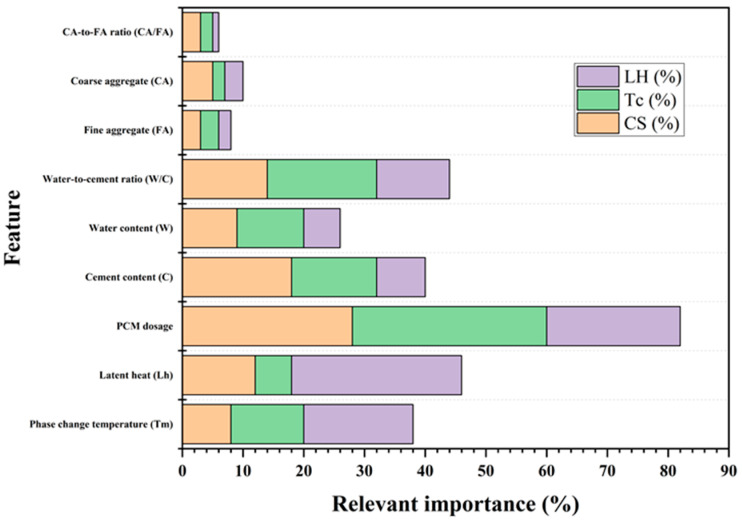
Analysis of feature importance.

As shown in Figure 26, feature importance was evaluated using CatBoost and XGBoost. PCM dosage, phase change temperature (Tm), and latent heat (Lh) are the dominant drivers. They jointly contribute over 70% of the importance. PCM dosage strongly affects compressive strength (CS) and thermal conductivity (Tc). Tm governs latent heat (LH) prediction and also influences Tc. Lh is central for LH and further contributes to CS. Cement content, water-to-cement ratio, and water content show moderate effects. Aggregate-related factors (CA, FA, CA/FA) are negligible. The results agree with Pearson correlation analysis. This reinforces the robustness and interpretability of the findings.

From an overall perspective, the models follow the trend: CatBoost > XGBoost > RF > SVR. The integration of swarm intelligence algorithms significantly enhanced both accuracy and stability, with WOA and FFA demonstrating the most prominent optimization effects [218]. Based on comprehensive evaluation across R^2^, RMSE, MAE, and MSE, the CatBoost-WOA model achieved the best performance in terms of prediction accuracy, error distribution, and generalization, making it the most promising candidate for modeling the thermal and mechanical properties. Metaheuristic optimization generally contributed positively to prediction performance [219]. Among the algorithms, WOA and GWO were especially effective in improving both precision and robustness across various models. CatBoost-WOA and XGBoost-WOA were identified as the most balanced and reliable model combinations across all three performance indicators.

### 3.6. Future Improvements

The proposed multi-model optimization approach demonstrated excellent performance in predicting the thermophysical and mechanical properties of cementitious composites. Particularly for key indicators such as thermal conductivity, latent heat, and compressive strength, the optimized models exhibited high predictive accuracy and stability. The integration of various swarm intelligence algorithms significantly improved the generalization capability and consistency of traditional machine learning models. Moreover, the variable selection framework and cross-validation scheme employed in this study provide a transparent and practical modeling pipeline, offering valuable guidance for researchers working on similar composite systems. The current study adopted a fixed data preprocessing pipeline and static hyperparameter spaces. Although multiple optimization strategies were applied, the sensitivity of model performance to different feature engineering methods, normalization techniques, and hyperparameter tuning has not been fully explored. Future research should consider the following improvements: Expand the dataset scope, especially by incorporating real-world and field-condition samples to improve model robustness. Integrate microstructural descriptors that are strongly correlated with heat conduction mechanisms to improve model generalizability and mechanistic interpretability.

## 4. Conclusions

This study presents a comprehensive exploration of the intelligent prediction of thermophysical and mechanical properties of cement pastes incorporating bio-based phase change materials. By combining bibliometric analysis with the multi-algorithm optimization of traditional machine learning models, this study reveals key research trends and thematic developments. In addition, it builds and validates high-accuracy models suitable for prediction and material design. The main conclusions are summarized as follows:

1. Based on a bibliometric analysis of 5928 core publications from 2013 to 2024, this study systematically reveals five major research hotspots—“*machine learning*,” “*thermal conductivity*,” *“energy efficiency,” “mechanical properties,”* and *“thermal energy storage”.* The bibliometric analysis not only identifies key research hotspots but also demonstrates the growing influence of machine learning in the cementitious materials field. These hotspots represent a shift from traditional studies focused on basic thermal properties to more complex, multifunctional applications.

2. PCM dosage, water-to-cement ratio, and the latent heat of BPCMs are identified as dominant factors influencing thermal conductivity, latent heat capacity, and compressive strength. A strong negative correlation is observed between BPCM dosage and both compressive strength and thermal conductivity. It indicates that while the incorporation of BPCMs improves thermal energy storage, it may lead to a decline in mechanical performance. This reflects a trade-off relationship and highlights the challenge of balancing multifunctional properties in composites.

3. Four mainstream predictive models were developed using 100 experimental samples to estimate the key performance metrics Tc, LH, and CS. By combining traditional machine learning models (e.g., CatBoost, SVR) with metaheuristic algorithms, this study demonstrated a significant improvement in predictive accuracy. Specifically, the CatBoost-WAO and SVR-GWO hybrid models outperformed conventional models in terms of robustness, showing R^2^ improvements of up to 44%. These results confirm that the hybrid approach not only enhances predictive performance but also stabilizes predictions across diverse datasets. This methodological advancement makes the models more reliable for material design.

4. The CatBoost-WOA model demonstrated the best performance, achieving R^2^ values of 0.927 (Tc), 0.955 (LH), and 0.944 (CS), with corresponding RMSEs of 0.0057 W/m·K, 1.84 J/g, and 2.91 MPa. Compared to unoptimized models, its prediction accuracy improved by 38.2%, 41.7%, and 44.4%, respectively. These results highlight its excellent robustness and practical adaptability for engineering applications.

5. GWO emerged as one of the most effective metaheuristic algorithms for hyperparameter tuning, particularly in small sample datasets. Its application led to substantial reductions in prediction error, with the RMSE decreasing by over 3.3 times for compressive strength prediction. This shows that GWO excels at fine-tuning models in constrained environments, making it an indispensable tool for predictive modeling in fields where high accuracy is crucial despite limited data. In compressive strength (CS) prediction, the coefficient of determination (R^2^) increased from 0.77 to 0.79. These results demonstrate GWO’s excellent capability in hyperparameter tuning, particularly in small-sample datasets.

6. The predictive models, through coordinated optimization, effectively manage the highly nonlinear relationships among input parameters such as PCM dosage and phase change temperature. This adaptability ensures the stability of the models even when parameters fluctuate. Their ability to generalize well across diverse datasets underscores their potential for use in real-world applications.

## Figures and Tables

**Table 1:** Optimization ranges and selected hyperparameters across models.

ML Model	Optimizer	Search Space	Population Size	Iterations	Best Configuration
SVR	GA	C ∈ [0.1, 100]; γ ∈ [0.001, 1]	30	100	C = 12–16; γ = 0.01–0.02
	PSO		30	100	C = 13–17; γ = 0.01–0.03
	WOA		25	120	C = 17–20; γ = 0.01–0.02
	GWO		30	150	C = 13–15; γ = 0.015–0.02
	FFA		20	100	C = 11–13; γ = 0.015–0.02
RF	GA	n_estimators ∈ [50, 500]; max_depth ∈ [3, 20]	30	100	n_estimators = 200–240; depth = 11–13
	PSO		25	120	n_estimators = 240–260; depth = 13–15
	WOA		30	150	n_estimators = 260–290; depth = 13–14
	GWO		20	100	n_estimators = 220–240; depth = 12–13
	FFA		25	120	n_estimators = 190–210; depth = 11–12
XGBoost	GA	learning_rate ∈ [0.01, 0.3]; n_estimators ∈ [50, 500]; max_depth ∈ [3, 15]	30	100	learning_rate = 0.07–0.09; n_estimators = 280–320; depth = 9–11
	PSO		25	120	learning_rate = 0.06–0.08; n_estimators = 300–330; depth = 8–10
	WOA		30	150	learning_rate = 0.04–0.06; n_estimators = 270–300; depth = 10–12
	GWO		20	100	learning_rate = 0.08–0.10; n_estimators = 330–360; depth = 9–11
	FFA		25	120	learning_rate = 0.05–0.07; n_estimators = 290–320; depth = 8–10
CatBoost	GA	learning_rate ∈ [0.01, 0.3]; depth ∈ [3, 15]; iterations ∈ [100, 1000]	30	100	learning_rate = 0.04–0.06; depth = 9–11; iterations = 580–620
	PSO		25	120	learning_rate = 0.05–0.07; depth = 8–10; iterations = 620–660
	WOA		30	150	learning_rate = 0.04–0.05; depth = 10–12; iterations = 680–720
	GWO		20	100	learning_rate = 0.06–0.08; depth = 9–11; iterations = 600–640
	FFA		25	120	learning_rate = 0.04–0.06; depth = 8–10; iterations = 560–600

Note: Due to the stochastic nature of metaheuristic optimization, specific values may vary slightly across runs but consistently fall within the indicated ranges.

## Data Availability

The original contributions presented in this study are included in the article. Further inquiries can be directed to the corresponding authors.
